# Optimization of Magneto-thermally Controlled Release Kinetics by Tuning of Magnetoliposome Composition and Structure

**DOI:** 10.1038/s41598-017-06980-9

**Published:** 2017-08-07

**Authors:** Behzad Shirmardi Shaghasemi, Mudassar Mumtaz Virk, Erik Reimhult

**Affiliations:** Institute for Biologically Inspired Materials, Department of Nanobiotechnology, University of Natural Resources and Life Sciences, Muthgasse 11, 1190 Vienna, Austria

## Abstract

Stealth (PEGylated) liposomes have taken a central role in drug formulation and delivery combining efficient transport with low nonspecific interactions. Controlling rapid release at a certain location and time remains a challenge dependent on environmental factors. We demonstrate a highly efficient and scalable way to produce liposomes of any lipid composition containing homogeneously dispersed monodisperse superparamagnetic iron oxide nanoparticles in the membrane interior. We investigate the effect of lipid composition, particle concentration and magnetic field actuation on colloidal stability, magneto-thermally actuated release and passive release rates. We show that the rate and amount of encapsulated hydrophilic compound released by actuation using alternating magnetic fields can be precisely controlled from stealth liposomes with high membrane melting temperature. Extraordinarily low passive release and temperature sensitivity at body temperature makes this a promising encapsulation and external-trigger-on-demand release system. The introduced feature can be used as an add-on to existing stealth liposome drug delivery technology.

## Introduction

The controlled assembly of liposomes and their investigation was pioneered in the 1960s^1^ and they were proposed as a potential drug delivery system in the 1970s^2, 3^. These nanocarriers provide a number of benefits, such as efficient and stable encapsulation and solubilization of compounds with varied physicochemical properties^4^, protection of compounds against degradation and prolonged circulation *in vivo* ^5, 6^. The inherent biocompatibility of liposomes is complemented by chemical modification with poly(ethylene glycol) (PEG) to achieve longevity during storage as well as long circulation time and low liver accumulation *in vivo*. PEG provides steric stabilization from adsorbing plasma proteins and aggregation of liposomes produced from saturated lipids, which has given rise to the expression “stealth liposomes”^6^. Nowadays, there are many approved liposomal drug formulations available and many others are in clinical trial, showcasing the success of stealth liposomes as a drug delivery system^6^.

A lower amount of drug can be used for therapy if drug release kinetics can be confined to and controlled at a specific location compared to current near systemic application of drug therapy; this radically reduces side effects such as toxicity and immune system reaction that hamper drug administration in general and in cancer therapy in particular^7, 8^. A general problem for drug delivery systems is the contradicting design requirements of stable encapsulation and efficient and fast release to control the drug concentration at the target site. The improvement achieved by accumulation of drug carriers at the point of release is partially offset if the release kinetics cannot be controlled such that the therapeutic concentration is released and maintained. An approach to circumvent too slow release has been to introduce liposomes with lipid compositions that change the membrane permeability and release rate in response to environmental triggers such as pH (triggered e.g. in endosomes)^9^ or temperature (e.g. triggered by elevated tissue temperature in tumors)^10^.

Temperature can be used to affect membrane permeability although liposomes can stably encapsulate hydrophilic compounds in both the gel phase and liquid phase. In the gel phase the lipid chain and lateral mobility is low, leading to a thick and rigid membrane, while in the liquid phase the lipid chain and lateral mobility is high, leading to a thin membrane with large area per lipid headgroup and large membrane fluctuations. At the membrane melting temperature (*T*_m_), defining the transition between the two phases, phase separation in the membrane gives rise to line defects that allow molecules to permeate through the membrane. This defect is due to the mismatch in chain extension and organization between the lipids at the phase boundary. If the temperature is increased to well above *T*_m_, the entire membrane is in the liquid phase, again presenting a membrane mostly impermeable to molecular and ion diffusion. The *T*_m_ can be controlled by lipid composition. Stealth liposomes with *T*_m_ close to body temperature suffer from leakage, while the release from vesicles with higher *T*_m_ is slow and inefficient. Relying on minor temperature differences *in vivo* for triggered release therefore invariably leads to an undesirable compromise with high passive release^11^. Still, compared to pH or biological cues, temperature has the advantage of being possible to control by external means, e.g. by incorporation of nanoparticles (NPs) that dissipate heat when exposed to external optical or magnetic fields^12–16^.

Incorporation of superparamagnetic iron oxide nanoparticles (SPION) for this purpose has the advantage that biological tissue has low magnetic susceptibility at frequencies in the 100 kHz range that can be used to heat SPION as small as a few nm in diameter^17^. Combining magnetic nanoparticles with vesicles to control their release properties has been implemented in many different ways with various degrees of success^11, 18^. If the strategies to incorporate NPs in the lumen, at the interface of a liposome membrane and in the membrane are compared, the latter is the most efficient^11, 18^. If the NPs are situated within the membrane, they can directly actuate it both mechanically (rotational or translational motion of the nanoparticles causing defects in the local membrane)^19, 20^ or thermally (heat transferred from the nanoparticles causing a phase transition or reorganization of the local membrane)^14, 16^. Incorporation of nanoparticles in the membrane is, however, challenging and easily leads to clustering, micelle formation^21^ and increased passive release^14, 16^. To be accommodated in a vesicle membrane nanoparticles have to be roughly smaller than the lipid membrane dimension (<6.5 nm)^22–24^, stabilized using a dense hydrophobic shell of irreversibly adsorbed ligands^16, 24^ with all free organic solvent removed. These are stringent demands, which so far have limited the possibility to produce and investigate rational design for stability and release parameters of such nanoparticle-actuated liposomes. In particular, the synthesis of monodisperse SPION which ends with SPION coated by an excess of oleic acid (OA) have to be re-grafted with a hydrophobic ligand that is irreversibly grafted in a dense shell around the iron oxide core and purified from OA. A weakly grafted shell and free or desorbing OA leads to particle aggregation, micelle formation and distortions to the liposome membrane causing uncontrolled passive release^14, 16^.

We recently introduced a one-step assembly method using solvent inversion to efficiently assemble and load nanoparticles into liposomes^24^. Here we report a breakthrough building on this platform, further simplified by applying sonication. The new method demonstrates efficient loading of both SPION and a water soluble compound into small unilamellar liposomes (∅ ~ 50 nm) using a protocol that allows us to generally and independently control nanoparticle fraction and lipid composition with different *T*_m_ and other membrane properties in the entire relevant range. This advance enables, for the first time, quantitative investigations of the influence of nanoparticle loading and membrane composition on release of encapsulated compound from magnetoliposomes using magnetothermal heating. Furthermore, we show how passive release is suppressed by detailed control over the stealth liposome assembly. The measurements of release kinetics were performed using alternating magnetic fields in the biomedically relevant frequency and field strength window; the water soluble fluorophore calcein was used to quantify release by the increase of fluorescence upon release from a self-quenching concentration inside the liposomes. Complete external control over the release kinetics from burst to pulsed gradual release is thereby demonstrated. Furthermore, the hypothesized mechanism for local magneto-thermal release is investigated and verified. The understanding of the release mechanism and its relation to magnetoliposome structure provides us with several ways to optimize the triggered release rate through the magnetoliposome composition, which are all demonstrated.

## Results and Discussion

**Figure 1:**
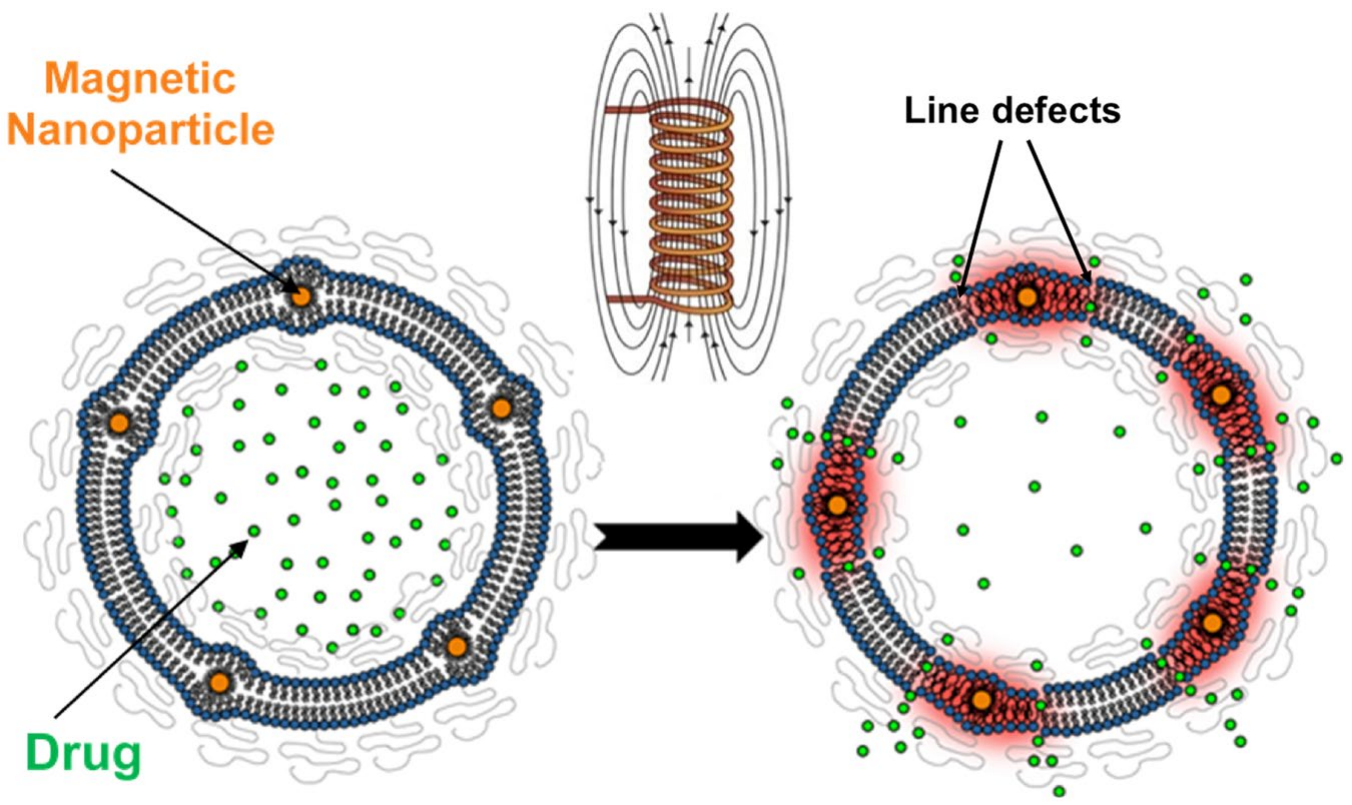
Local heat generation from SPION through Néel’s relaxation produces a line defect in the membrane separating melted (liquid) from solid domains.

The embedded SPION are expected to produce heat through Néel relaxation in an alternating magnetic field at 228 kHz due to their small size (∅ = 3.9 nm)^17^. The heat produced locally at the nanoparticle is dissipated through the surrounding lipid membrane. We hypothesize that the lipids near each SPION can reach a temperature much higher than the surrounding bulk. In fact, Dong *et al*. have demonstrated that the local temperature around the SPION can be significantly higher than the bulk temperature when the particles are subject to an alternating magnetic field (AMF) locally heating the SPION through Néel relaxation^25^. If the lipid membrane has a melting temperature, *T*_*m*_ above the ambient bulk temperature, the local high temperature induces a local phase separation between a melted liquid membrane and a gel phase membrane in the rest of the liposome (Fig. 1). The line defect between the two phases has high permeability for the encapsulated compound and thereby provides a mechanism for controlling magnetoliposome membrane permeability. Triggered release should continue only as long as the AMF produces the local heat gradient around the SPION in the membrane and should stop almost instantly by removing the AMF due to the fast heat dissipation from membranes in aqueous environments^18, 26^. The effect is local and does not require increasing the bulk temperature. Despite that local heating is often invoked as the explanation for nanoparticle-triggered release through electromagnetic fields, it has been disputed in theoretical and experimental studies and remains controversial^26–28^. Detailed control over monodisperse particle size and vesicle composition, as presented in this study, is required for experimental investigations of the heat-triggered-release hypothesis. The vesicle composition was varied by using four different phosphocholine lipids that share the same headgroup but have different fatty acid chain lengths and saturation: 1-palmitoyl-2-oleoyl-*sn*-glycero-3-phosphocholine (POPC, *T*_*m*_ = −2 °C), 1-myristoyl-2-palmitoyl-*sn*-glycero-3-phosphocholine (MPPC, *T*_*m*_ = 35 °C), 1,2-dipalmitoyl-*sn*-glycero-3-phosphocholine (DPPC, *T*_*m*_ = 41 °C) and 1,2-distearoyl-*sn*-glycero-3-phosphocholine (DSPC, *T*_*m*_ = 55°C). The difference in chain length leads to different membrane *T*_*m*_ of the respective liposomes. We hypothesize that a lower *T*_*m*_ can lead to higher susceptibility to thermally induced local phase separation and a correlating increase in permeability of magnetoliposomes.

Magnetoliposomes were formed by a novel route based on solvent inversion^24^ followed by an added step of bath sonication for additional sizing and homogenization. The size and stability of the magnetoliposomes were investigated to ensure the formation of a monodisperse distribution of unilamellar liposomes. The efficiency and the length of the sonication step is dependent on the sonicator power, size, as well as the sample position, and it can also vary with the wear on the sonicator. Magnetoliposomes with the same size distribution could reproducibly be produced for the same lipid composition using the same sonicator parameters; the parameters used for all magnetoliposomes reported here are descried in the Methods section.

**Figure 2:**
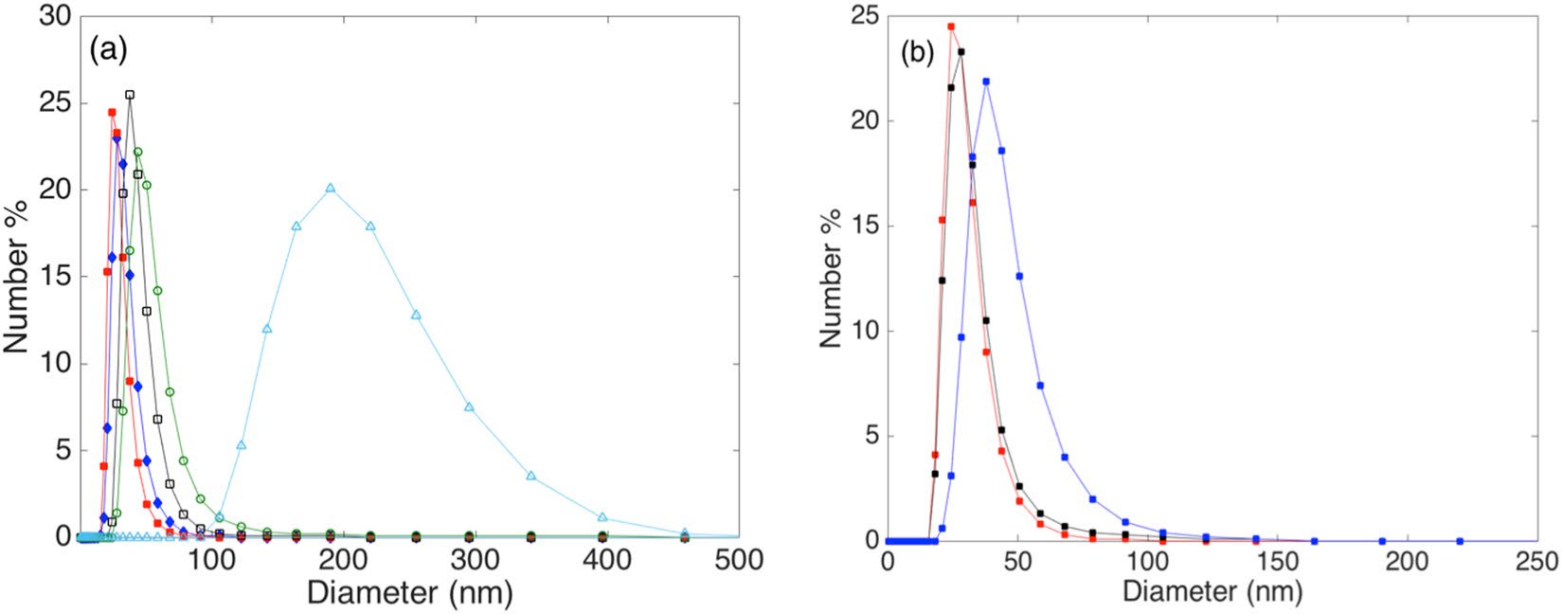
(**a**) Size distributions of MPPC liposomes containing 2 wt% (), 4 wt% (), 6 wt% (), 8 wt% (), 10 wt% () SPION after formation. (**b**) Size distribution of MPPC with 4 wt% SPION immediately after formation (red), after 1 week (black) and 11 months after their formation (blue).

Figure 2 demonstrates that narrow, monomodal size distributions were recorded by dynamic light scattering (DLS) for MPPC magnetoliposomes, with no indication of smaller or larger aggregates being present. An increasing average vesicle size was observed when the nanoparticle weight fraction was increased. The size distributions of magnetoliposomes with SPION fractions of up to 4 wt% to lipid mass was unchanged after 11 months of storage, and no change in coloration, scattering or precipitates could be observed visually or by DLS (see Figs 2 and SI-1a,b), indicating perfect colloidal stability of magnetoliposomes over (at least) this time period. Monodisperse and long-time stable magnetoliposomes could be formed by this method for all saturated lipids (MPPC, DPPC and DSPC) at SPION concentrations up to 4 wt%. This indicates that sonication can circumvent the problem of interdigitation during tetrahydrofuran (THF) removal that prevents formation of liposomes with high *T*_*m*_ by pure solvent inversion, which was previously reported^24^. Above 4 wt% SPION, vesicle suspensions deteriorated over 1 week with visible precipitation (see Fig. SI-1a). This indicates that incorporation of very high contents of SPION using this method likely results in encapsulation of aggregates in the membranes^21, 24^. However, investigations of magnetoliposomes by transmission electron microscopy (TEM) stabilized in trehalose or by cryo-TEM (see Figs SI-2 and 3) generally showed the SPION well dispersed in the lipid membrane. This was true at low concentration of SPION, for which mostly only single SPION per vesicle were observed, as well as at high concentration of SPION, for which a larger number of non-aggregated SPION per vesicle could be observed. TEM on large vesicles before the sonication step^24^ has shown that the SPION are dispersed in liposomes already before the sonication. A larger number of particles per vesicle is expected for vesicles with larger size, which is also observed. The absence of aggregates of nanoparticles in TEM for high SPION concentrations could be due to that preparation of samples for TEM favors imaging of vesicles compared to smaller aggregates that could be washed away during sample preparation and therefore not found.

**Figure 3:**
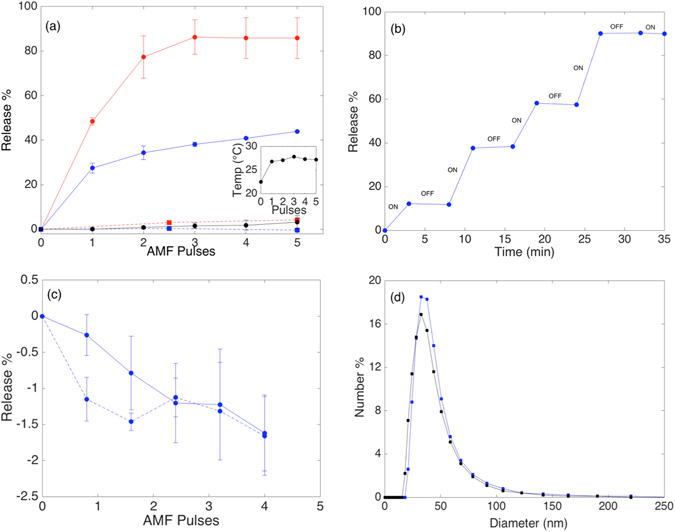
(**a**) Calcein release kinetics of MPPC magnetoliposomes (*T*_*m*_ = 35 °C) with 2 wt% SPION (blue), 4 wt% SPION (red) and without SPION (black) as function of exposure time to AMF. The dotted lines represent the respective passive release measured during the same time with 5-min equilibration time between AMF pulses. The error bars show the standard error of the mean between two different measurements of two independent samples. The inset shows the bulk temperature of the sample after each 2-min pulse. (**b**) The immediate stop of release as the AMF was switched off is demonstrated by remeasuring the fluorescence before every AMF pulse for DPPC vesicles (*T*_*m*_ = 41 °C) containing 4 wt% SPION. The duration of the AMF pulses was 3 min and the time between each pulse was 5 min. (**c**) POPC (*T*_*m*_ = −2 °C) liposomes incorporating 4 wt% SPION exposed to 4-min AMF pulses (solid line) and to no field (passive release; dashed line) both show absence of release since the membrane is already in the liquid phase at room temperature. The liposomes are already at room temperature above their *T*_*m*_ and show neither significant passive nor triggered release. The negligible trend to increasingly negative release % values is due to photobleaching of the background fluorescence signal for each subsequent measurement. The averages and the standard errors of the mean of two measurements of two independent samples are shown. (**d**) The hydrodynamic size distribution (average of three measurements) of MPPC magnetoliposomes with 4 wt% SPION before (blue) and after (black) actuation.

The concentration of SPION in the membrane strongly affected the susceptibility of the liposomes to release encapsulated calcein by exposure to alternating magnetic field. Figure 3a shows the effect of changing the SPION concentration from 2 to 4 wt% on release efficiency. The alternating magnetic field leads to a clear release of calcein at a pulse length of 2 min. For 4 wt% SPION in MPPC liposomes (*T*_*m*_ = 35 °C) the first 2-min pulse releases 48.4% of the encapsulated content. Only three pulses are required to release the maximum amount of encapsulated content, which averaged ~90% for the MPPC liposomes. When 2 wt% SPION are incorporated, only 28% of calcein is released in the first pulse and the total release seems to saturate slowly to a lower value than for 4 wt%. After 5 pulses the accumulated release is still lower than ~44% of the total amount of encapsulated calcein. Thus, the reduction in SPION weight fraction to half seems to reduce the total amount of calcein that can be released as well as the rate of release. The same result was observed for DPPC (Fig. SI-4), i.e. the rate of release per pulse is also halved. However, when the calcein released by each pulse is normalized by the maximum amount of calcein released by magnetic trigger for the same sample, then this fraction is independent on SPION concentration. These results strongly imply that only a fraction of mangetoliposomes contribute to the release for 2 wt% SPION, but that the rate of release of each magnetoliposome/SPION that contributes to release is equal for 2 wt% and 4 wt% SPION. Increasing the fraction of nanoparticles beyond 4 wt% affected the integrity of the encapsulation, leading presumably to clustering of nanoparticles, and therefore more inhomogeneous distribution of SPION between magnetoliposomes. The detrimental effect of this on the release efficiency can be observed when the wt% of SPION is changed from 2 to 8wt% in Figs SI-4 and SI-5a,b. Also the passive release was increased. We thus limited further triggered release studies to 2 and 4 wt% SPION.

It is important for the validity of the previous comparison as well as for applications that there is no passive release and that the phase transition can be reached without increasing the bulk temperature above *T*_*m*_. In the inset of Fig. 3a we observe that the sample temperature stays constant at 27 °C after the initial pulse, which is well below *T*_*m*_ = 35 °C. Furthermore, Fig. 3a shows negligible passive release over the time of the triggered release experiment.

Release through mechanical distortion of the membrane has been reported^19, 20^. This requires that Brownian relaxation by rotation of the SPION in the membrane takes place^17^, but it can be excluded for 3.9 nm SPION in the viscous membrane environment at 228 kHz^17, 20^. Furthermore, when magnetic release has been reported through mechanical distortion the process has appeared to cause irreversible damage to membrane integrity and high passive release after the magnetic fields was switched off was reported^19^. In contrast, in our case, the calcein release ceased when the AMF was switched off and no further release was observed after an AMF pulse until the sample was exposed to the next pulse. The samples were equilibrated for 5 min between AMF pulses during which the calcein fluorescence was measured and no additional release was recorded between AMF pulses. This is shown in Fig. 3b for DPPC liposomes containing 4 wt% SPION, which are the magnetoliposomes with the lowest *T*_*m*_ in our study and therefore the most prone to release after the field has been switched off. These results demonstrate that as soon as the alternating magnetic field is removed the release is as negligible as the passive release. There is also no release observed for MPPC liposomes without NPs subject to the same AMF pulses (Fig. 3a), i.e. the observed release kinetics can exclusively be attributed to AMF actuation through the incorporated SPION. Finally, a test of whether release occurs through membrane distortion or local heating leading to local phase separation at the *T*_*m*_ of the liposome membrane can be tested by applying the same test to POPC magnetoliposomes, which are already in the liquid phase. This test is shown in Fig. 3c and shows that neither passive nor triggered release occurs for liposomes already in the liquid phase. The vesicle size also remained constant during actuation, as showed by DLS measurements of the hydrodynamic diameter of magnetoliposomes before and after AMF exposure (Fig. 3d).

Together, these results strongly support the mechanism of local release hypothesized above (Fig. 1), which is dominated by heat generated by the SPION through Néel relaxation^17^ and leading to local phase separation of lipids along a radial temperature gradient. The line defect at the lipid phase boundary around the actuated SPION that is introduced by the localized magnetic heating immediately disappears when the field is switched off as the membrane cools down to bulk temperature (below *T*_*m*_) and changes its phase from liquid to gel. Release is therefore achieved through reversible, local change of the membrane permeability without affecting overall vesicle structure. The constant relative rate of release across the samples normalized per contributing SPION also supports a local origin of the release mechanism. It is important to realize that a temperature gradient and not the high temperature at the SPION itself increases the permeability, since heating of the entire membrane to the liquid phase would close the defect that enables the release and lead to the same result of no permeation as observed in Fig. 3c.

**Figure 4:**
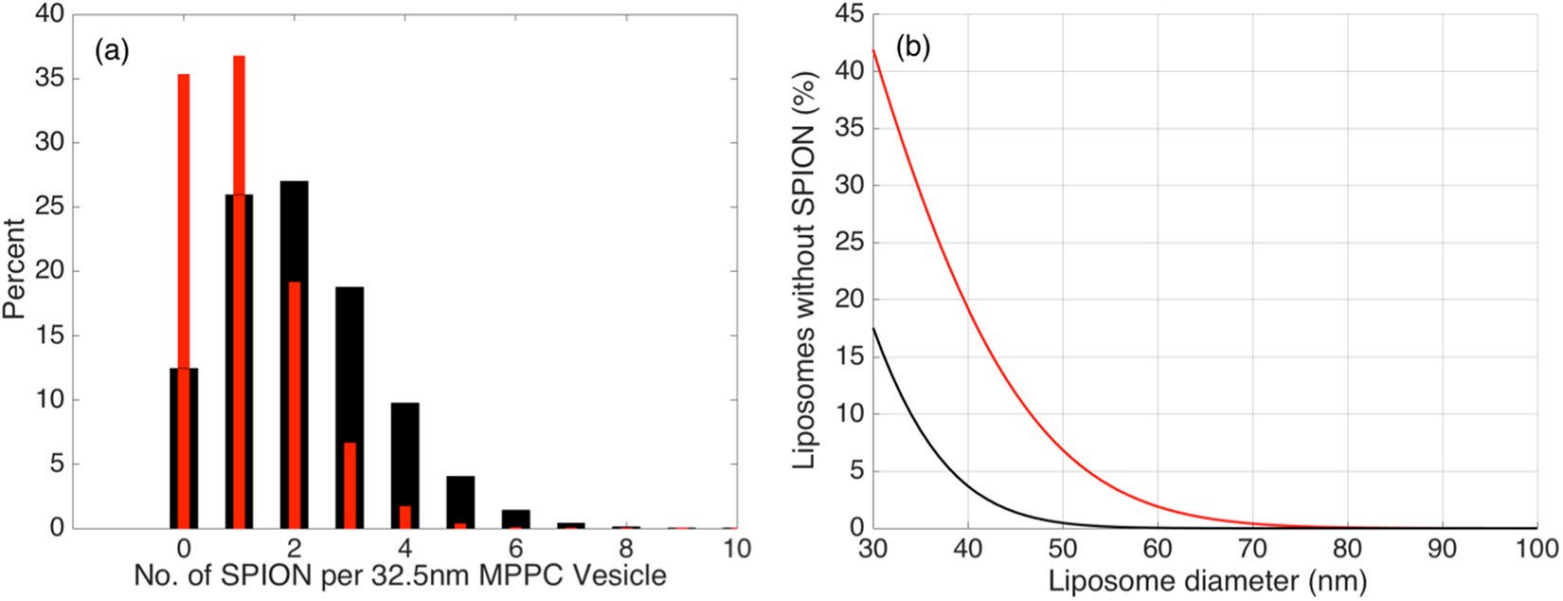
(**a**) Histogram of the expected percentage of 32.5 nm MPPC liposomes (measured average size of magnetoliposomes) containing a certain number of SPION comprising 2 wt% (red) or 4 wt% (black) SPION. (**b**) A fraction of liposomes will contain no nanoparticles when the nanoparticles are stochastically distributed between liposomes. The percentage of liposomes not containing any SPION as function of liposome size has been calculated according to the Poisson distribution when the average liposome comprises 2 wt% (red) or 4 wt% (black) SPION.

The local, nanoscale nature of the release has the consequence that the distribution of SPION and thereby pores that can be formed across the vesicle population becomes important. As observed in Fig. 3a, only approximately half of the 2 wt% SPION liposome cargo was released upon actuation, while liposomes with 4 wt% SPION released 90% of their cargo. This observation can be explained if the lower SPION concentration leads to fewer liposomes that contain at least one SPION. If all vesicles have at least one SPION in the membrane, different numbers of SPION per vesicle should not affect the total amount of encapsulated compound that is released even if it affects the rate by which the release occurs. For our monodisperse SPION we measured the average mass of single particles by thermogravimetric analysis (TGA) **(**see Fig. SI-6). Using the liposome size distribution recorded by DLS and known values for the lipid Mw and head group area^29^ the number of SPION per vesicle can be calculated as *N*_*SPION*/*ves*_ = (*m*_*SPIONtot*_ *N*_*lip*/*ves*_)/(*m*_*SPIONtot*_ *N*_*lip*_) where *m*_*SPIONtot*_ is the total mass of SPION added to the sample, *N*_*lip*/*ves*_ is the number of lipids per vesicle depending on the size of the vesicle, *m*_*SPION*_ is the mass of a single SPION and *N*_*lip*_ is the total number of lipids. The average number of SPION per MPPC liposomes with a diameter of 32.5 nm in diameter (corresponding to the vesicles measured in Fig. 3d) calculated in this way is 2.1 and 1.05 for 4 wt% and 2 wt% SPION, respectively (see SI-7). This average number is expected to be distributed across the liposome population according to the Poisson distribution and we can calculate the probability of a vesicle containing a given number of SPION (see SI-7). Figure 4a demonstrates the difference in the distribution of the number of SPION per vesicle for liposomes 32.5 nm in diameter with 2 wt% or 4 wt% SPION. For 2 wt% SPION, 35.3% of all liposomes do not contain any SPION, which is close to the fraction of calcein that was not released for such MPPC magnetoliposomes (Fig. 3a). For 4 wt% SPION, more than 85% of all vesicles of this size are expected to contain at least 1 nanoparticle, which is in good agreement with the data reported in Fig. 3a. Although, a rough estimate, the limited number of particles per nanoscale liposome and the statistical distribution of SPION across the liposome population is in very good quantitative agreement with the release data. The presented magnetoliposomes produced by our solvent inversion-sonication method have a small diameter. It is worthwhile to consider that as the membrane area of a liposome increases with size, the likelihood of finding at least one SPION acting as a gate in the membrane increases fast. Figure 4b shows the expected percent of liposomes containing zero SPION as a function of liposome size for magnetoliposomes comprising on average 2 wt% and 4 wt% SPION. Clearly, all liposomes will eventually contain nanoparticles and be susceptible to magnetic release as the vesicle size increases. However, for vesicles with diameters smaller than 70 nm a significant fraction will not contain any SPION (see SI-8,9). Attempts to image SPION in small liposomes at these concentrations by TEM, where only a small cross-section of the liposome can be observed, therefore only rarely resulted in the imaging of SPION within a liposome structure such as in Fig. SI-3.

**Figure 5:**
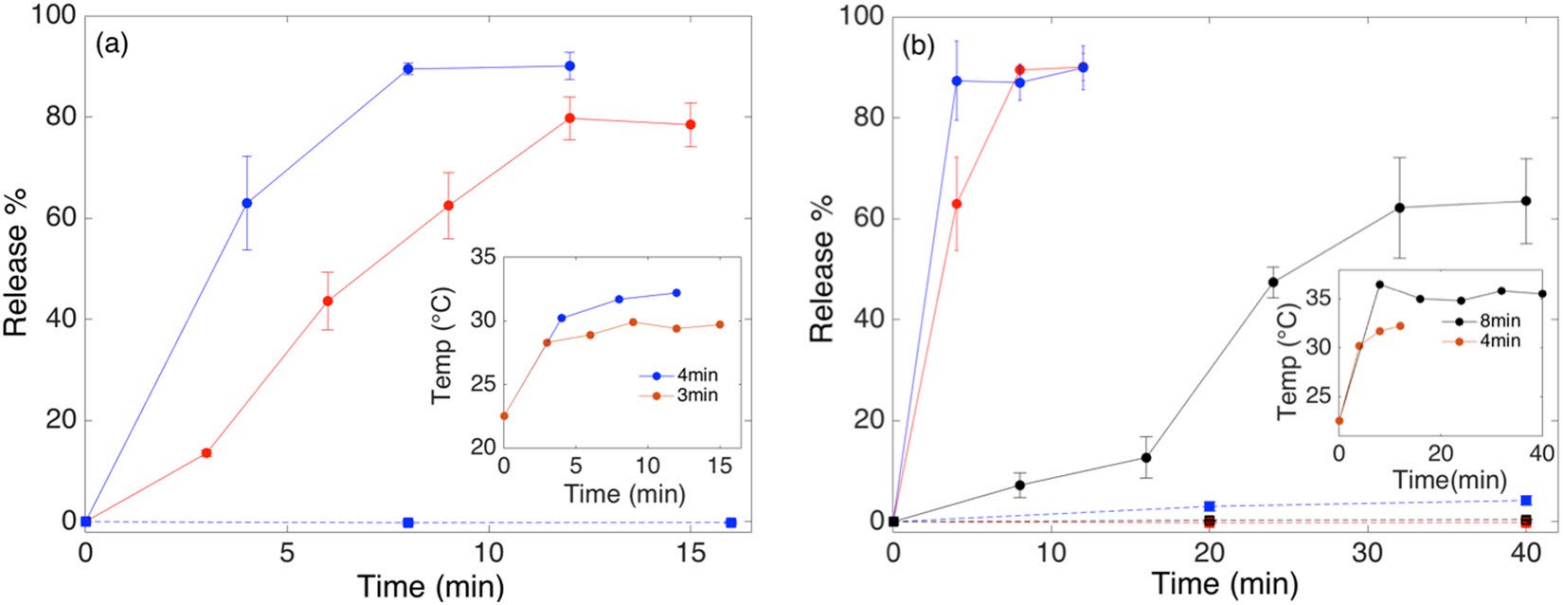
(**a**) Calcein release kinetics of PEGylated DPPC (*T*_*m*_ = 41 °C) vesicles comprising 4 wt% SPION exposed to 3 min AMF pulses (solid red) and 4 min AMF pulses (solid blue). The averages and standard errors of the mean are shown for four different measurements on two independent samples. (**b**) Release kinetics for 4 wt% SPION PEGylated magnetoliposomes exposed to 4 min AMF pulses for MPPC (*T*_*m*_ = 35 °C) (solid blue) and DPPC (solid red) and 8 min AMF pulses for DSPC (*T*_*m*_ = 55 °C) (solid black). The error bars show the standard errors of three (blue), four (red) and two (black) different measurements on two independent samples. The dotted lines represent the passive release measured during the same time for the sample of the respective color. The insets show the bulk temperature during actuation of the respective sample, which is attributed to Joule heating of the solution.

With the demonstrated release mechanism, we expect that each SPION effectively produces a defect boundary that is constant in size over the period of actuation as it is a function of a local temperature gradient. Thus, the release rate for a given magnetoliposome population is constant during actuation as observed in Fig. 3, but the amount of released calcein per pulse should be proportional to the pulse length since the temperature gradient stays the same (see insets Fig. 3a). Figure 5a shows a comparison of DPPC magnetoliposomes with 4 wt% SPION for different pulse durations. The amount of calcein released per pulse increases with pulse duration as expected. However, a linear scaling with the pulse duration is not observed, since for example an accumulated pulse time of (2 × 3) = 6 min releases less calcein than a single 4-min pulse. This indicates a lag phase upon AMF exposure before either the local temperature is sufficient high, or until the lipid reordering has taken place to establish the high-permeability line defect.

The inference of a lag phase that tentatively is a result of the time to establish a sufficiently high local temperature to create a domain of liquid phase lipids around the SPION, indicates that the choice of membrane *T*_*m*_ also will influence the release per pulse with all other parameters equal. Three different single lipid species magnetoliposomes were used to test this hypothesis: MPPC (*T*_*m*_ = 35 °C), DPPC (*T*_*m*_ = 41°C) and DSPC (*T*_*m*_ = 55 °C). Figure 5b shows the release kinetics for magnetoliposomes incorporating 4 wt% SPION when they are exposed to 4-min (for MPPC (blue) and DPPC (red)) and 8-min (for DSPC (black)) AMF pulses. One pulse of 4 min is sufficient to achieve maximum release for MPPC whereas two subsequent pulses of the same length are required to achieve maximum release for DPPC. AMF pulses of 4 min duration also did not lead to measurable release for DSPC magnetoliposomes; the slow release obtained for pulses of 8 min is shown instead as comparison (see Figure SI-10 for the release for pulses of 12 min). Clearly, the expected trend of the requirement of longer pulses for the same release when membrane *T*_*m*_ is increased is observed.

For all pulse lengths, the sample temperature was raised to a saturation value after one pulse (insets Fig. 5). The temperature increases were the same for samples without SPION and are attributed to Joule heating. The saturation temperature increased slightly with pulse length, but in all cases it was far below the respective lipid phase transition temperature and below body temperature.

**Figure 6:**
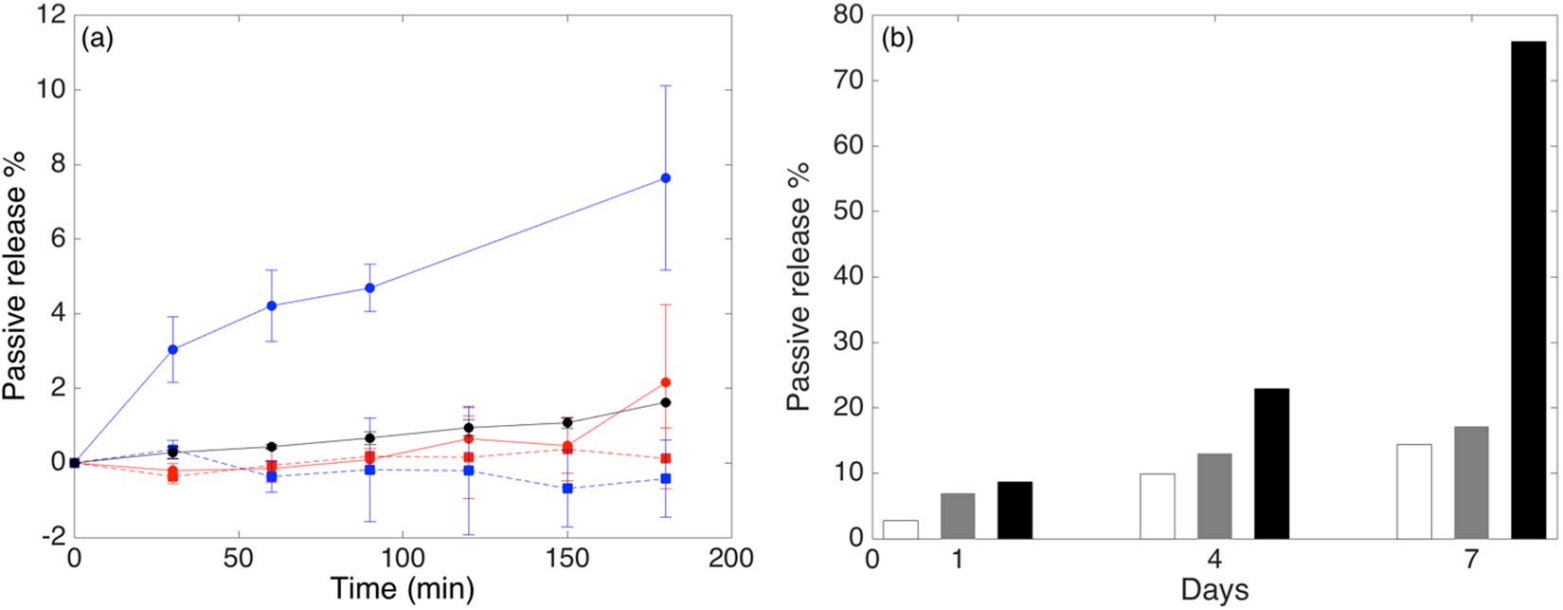
(**a**) Passive release for PEGylated magnetoliposomes containing 2 wt% (dotted) and 4 wt% (solid) SPION for liposomes comprising MPPC (blue), DPPC (red) or DSPC (black) lipid. The averages and standard errors of the mean are shown for measurements on two independent samples. (**b**) The passive release over one week for PEGylated DPPC liposomes: (white) kept at high vacuum for 72 h during lipid drying for chloroform removal, (gray) with 4 wt% SPION and chloroform removal using high vacuum for 72 h, (black) with 4 wt% SPION without chloroform removal using high vacuum.

Negligible passive release was observed for all samples during a time frame of several hours (Fig. 6a). There is a weak tendency that lower *T*_*m*_ lipids have higher passive release than higher *T*_*m*_ lipids, which can only be observed for the 4 wt% SPION samples. However, there is significant passive release observed over one week for magnetoliposomes prepared by standard protocols for lipid drying (Fig. 6b, black bars). This passive release could be reduced greatly by keeping the lipids at high vacuum for 72 h before mixing them with SPION. Figure 6b shows the passive release reduced from 76% to 17% over the course of 1 week when chloroform was removed from the lipid by applying high vacuum for 72 h. Although the presence of chloroform was not verified in the samples, it is likely that residual chloroform could only be sufficiently removed by the additional high vacuum step. Physical properties of lipid membranes are very sensitive also to trace amounts of organic solvent. Residual chloroform in the membrane may influence the fluidity and lipid order, which can enhance membrane permeability and lead to faster passive release^30^. We note that the passive release of <20% over 1 week for an externally triggered release system provides the possibility to release hydrophilic compounds with complete control over release kinetics over extended periods of time.

## Conclusions

We have demonstrated that solvent inversion-sonication is an efficient method to produce hydrophobic SPION-containing liposomes loaded with water soluble compounds for lipids membrane compositions with fully tunable melting temperature. It is possible to control SPION concentration to the maximum amount providing colloidal stability, which with this method was shown to be 4 wt% regardless of lipid composition. By carefully removing all chloroform and THF the colloidal stability of the magnetoliposome system is at least one year and passive release of encapsulated calcein is minor over weeks. By this universal approach we demonstrated unprecedented control over the release kinetics from magnetoliposomes, which was tuned from almost complete release within one single short AMF pulse to the repeated release of small doses. The release mechanism underpinning these achievements was shown to be in full agreement with the expectations of release occurring for membrane defects due to magneto-thermally induced lipid phase separation around individual SPION embedded in the liposome membrane. We expect that such detailed control over structure and function of nanoparticle-membrane hybrid materials will find direct application in drug delivery and other encapsulation and release applications, as well as inspire further design of magnetically controlled smart materials.

## Methods

### Synthesis of palmityl-nitrodopamide (PNDA)

Nitrodopamine (NDA) was synthesized according to literature with slight modifications (see SI-11a–f)^31^. Palmityl-nitrodopamide (PNDA) was synthesized by coupling of nitrodopamine (NDA) with palmityl-NHS (see SI-12a,b) in dimethylformamide (DMF) and purified by solvent extraction (0.1 M HCl and DCM) and recrystallized in ethyl acetate and chloroform (1:1) (see SI-13a–d).

### Synthesis of monodisperse iron oxide nanoparticles

Oleic acid - coated superparamagnetic iron oxide nanoparticles (OA-SPION) were synthesized via thermal decomposition of iron pentacarbonyl^32^. All materials were used as received without further purifications. In a typical procedure 1 ml of iron pentacarbonyl was quickly injected into a N_2_-saturated solution of 50 ml dioctylether containing 4 ml oleic acid at 100 °C. The temperature was then gradually raised to 290 °C with a ramp of 3 K/min and held for 1 h. The as-synthesized iron oxide nanoparticles were subsequently cooled to room temperature, precipitated from excess of EtOH and collected with external magnet. The particles were washed 5 times with ethanol and centrifuged at 5000 rpm for 1 minute to remove excess of oleic acid and dioctylether. The diameter of the resulting highly monodisperse and monocrystalline iron oxide particles was 3.9 ± 0.3 nm, calculated by the freeware Pebbles^33^ based on TEM images (see Figure SI-14a).

### Ligand exchange on oleic acid capped iron oxide nanoparticles (OA-SPION)

The mainly physisorbed OA has to be displaced by an irreversibly grafted hydrophobic ligand to obtain SPION grafted with a stable hydrophobic shell. To achieve this, coating of OA-SPION with PNDA through ligand exchange was done as previously reported^31^. In brief, 200 mg as-synthesized SPION were purified by repeated pre-extraction in hot MeOH containing 1 mM oleic acid as stabilizer before ligand exchanged in a mixture of 150 mg PNDA in DMF:CHCl_3_:MeOH (6:3:1) and sonication for 3 h (Elma/Germany, Transsonic T 460(14 × 24 × 7 cm), power 100%, Frequency 37 Hz) under nitrogen atmosphere. Newly capped SPION were evaporated to the DMF fraction, precipitated by adding excess MeOH and collected via magnetic decantation. The particles were purified by threefold extraction in hot MeOH to remove excess of PNDA. Resulting SPION were post-coated with 100 mg PNDA in minimal 2,6-lutidine for 48 h at 50 °C under nitrogen atmosphere, evaporated to dryness and purified by hot MeOH extractions and magnetic decantation. SPION were then lyophilized from tetrahydrofuran (THF):H_2_O (1:5) and dispersed in THF at concentration of 1 mg/mL and stored at −20 °C. The particles remain completely dispersed in THF without any precipitation after >2 years.

### Preparation of calcein-loaded liposomes with SPION incorporated in the membrane (magnetoliposomes)

5 mg of lipid (POPC, MPPC, DPPC or DSPC) and 1,2-dioleoyl-sn-glycero-3-phosphoethanolamine-N-[methoxy(polyethylene glycol)2000 Da] (PEG(2)-PE) (95:5 wt%) were added to a 5 ml round bottom flask with CHCl_3_, dried using rotavapor for 30 min (200 mbar, 60 °C) and dried under high vacuum (0.05 mbar) overnight. 200 µL of SPION from stock solution in THF were added to the lipid cake. The volume of SPION solution was kept constant while the concentration of SPION was varied to achieve the desired weight percent of SPION. For example, 4 wt% SPION required 1 mg/ml SPION concentration in THF. The flask containing lipid and SPION in THF was connected to a rotary evaporator and submerged in a water bath (*T*_*bath*_ > *T*_*m*_, where *T*_*m*_ is the transition temperature between the gel and liquid crystal phase of the lipid membrane) at 1 atm and stirred for 30 seconds to mix the lipid and SPION thoroughly. 1 ml of preheated (60 °C) and filtered PBS buffer (10 mM phosphate-buffered saline, 137 mM NaCl, 2.7 mM KCl at pH 7.4) containing 10 mM calcein was added to the lipid-SPION suspension in THF. Calcein at this concentration is above the self-quenching concentration and does not display strong fluorescence (see SI-15). Alternatively, the suspension of lipid and SPION in THF could be added dropwise to the buffer. In both cases, the flask containing a turbid solution was immersed in an ultrasonic bath (Transsonic T 460, power 100%, Frequency 37 Hz) and sonicated for 30 minutes (*T*_*bath*_ > *T*_*m*_ *of lipid*) to form a translucent solution. 1 ml of PBS containing 10 mM calcein was added to the solution to dilute the sample to 2.5 mg/ml of lipids. Gel filtration was used to separate the sample from non-encapsulated calcein and residual THF. Superdex 75 columns (composite of cross-linked agarose and dextran) were used with PBS as eluent, and the sample was collected using a Biological Duoflow chromatography system. An injected 2 ml sample was collected as 3–4 fractions of the same volume. The collected samples of magnetoliposomes encapsulating a self-quenched concentration of calcein were almost colorless (see Figure SI-16). A quantitative transfer of SPION to lipid ratio is obtained in this size range through the preparation method as previously demonstrated by UV/Vis spectroscopy (optical density measurements) and thermogravimetric analysis^24^, since the entire sample is collected and no precipitation takes place.

### Release Experiments

Passive and triggered release was quantified by measuring the calcein fluorescence of the samples over time and as function of exposure to pulsed alternating magnetic fields. The fluorescence of released calcein is not quenched due to the bulk concentration being below the self-quenching concentration of calcein even after complete release. The release can therefore be determined quantitatively by measuring the relative increase in fluorescence intensity compared to the same sample after lysis of the liposomes using equation 1:

1 $$Release\, \% =\frac{{I}_{i}-{I}_{AMF/PR}}{{I}_{i}-{I}_{tot}}$$

where *I*_*i*_ is the initial fluorescence intensity measured immediately after column purification, *I*_*AMF*_ is the fluorescence intensity measured after the sample was subjected to AMF and *I*_*PR*_ is the fluorescence intensity measured at different times without applying AMF in order to calculate passive release, and *I*_*tot*_ is the fluorescence intensity measured for complete release of all calcein by adding Triton X100 to lyse the liposomes. The fluorescence intensity was measured using fluorescence spectrophotometer (PerkinElmer Instruments, LS 55) at an excitation wavelength of 495 nm and emission wavelength of 515 nm. When required, the sample was diluted to be within the optimal working range of photo detector. Magnetoliposomes were subjected to radiofrequency alternating magnetic fields using an Easyheat setup (Ambrell) to investigate triggered release. The AMF was induced by an AC current of 439 A running through a coil of 3.7 cm at a frequency of 228 kHz, which yields a field strength of 94.5mT in the center of the coil where the sample is placed.

### Transmission Electron Microscopy

TEM studies were performed on a FEI Tecnai G2 20 transmission electron microscope operating at 160 kV. Nanoparticle samples were prepared by dropping THF suspension of PNDA coated particles (1 mg/ml) onto 300-mesh carbon-coated copper grids and subsequently evaporating the solvent in air. Size distributions were evaluated using Pebbles software^33^.

### Thermogravimetry Analysis

Thermograms were recorded on a Mettler-Toledo TGA/DSC 1 STAR System in the temperature range 25–650 °C with a ramp of 10 K/min in synthetic air (O_2_). For each measurement 5 mg of samples used and the mass lost were evaluated at 650 °C.

### Nuclear Magnetic Resonance Spectroscopy

^1^H and ^13^CNMR spectra were collected on a Bruker DPX operating at 300 MHz using TMS as an internal standard.

### ElectroSpray Ionization Time-of-Flight (ESI-ToF) Mass Spectrometry

Mass spectra of nitrodopamine (NDA) were measured on a Bruker Autoflex speed. Matrix (dithranol in THF (20 mg/mL)) was mixed with the sample (without additional salt) and dropped on the sample holder.

### Infrared Spectroscopy

Mid-infrared (IR) powder spectra of the samples were collected using a Bruker Tensor 37 FTIR spectrometer with Bruker Platinum Diamond single-reflection ATR equipment at a resolution of 4 cm^−1^ by averaging 32 scans.

### UV-VIS Spectroscopy

UV-VIS absorption spectra were collected at a scan speed of 400 nm/min on a Hitachi UV-2900 spectrophotometer.

### Dynamic Light Scattering

Characterization of the Brownian size of the nanoparticles and magnetoliposomes were performed on Malvern Instruments Zetasizer Nano-ZS instrument. The hydrodynamic size of PNDA–capped SPION in THF (1 mg/ml) and magnetoliposomes in tris buffer (1 mg/ml of lipid) measured at room temperature.

## Electronic supplementary material

Supporting Information
