# Author Correction: Optimization of Magneto-thermally Controlled Release Kinetics by Tuning of Magnetoliposome Composition and Structure

**DOI:** 10.1038/s41598-018-27849-5

**Published:** 2018-06-21

**Authors:** Behzad Shirmardi Shaghasemi, Mudassar Mumtaz Virk, Erik Reimhult

**Affiliations:** Institute for Biologically Inspired Materials, Department of Nanobiotechnology, University of Natural Resources and Life Sciences, Muthgasse 11, 1190 Vienna, Austria

Correction to: *Scientific Reports* 10.1038/s41598-017-06980-9, published online 07 August 2017

The Supporting Information file that accompanies this Article contains errors in SI-7, ‘The average mass of single SPION and number of SPION in Magnetoliposomes’,

“$${\rm{core}}\,{\rm{mass}}\,{\rm{of}}\,{\rm{single}}\,{\rm{SPION}}=5.024\frac{{\rm{g}}}{{10}^{21}{{\rm{nm}}}^{3}}\times 31.059\,{{\rm{nm}}}^{3}=1.627\times {10}^{-19}{\rm{g}}$$

$${\rm{Shell}}\,{\rm{mass}}=\frac{34}{66}\,{\rm{total}}\,{\rm{mass}}\,{\rm{of}}\,{\rm{single}}\,{\rm{SPION}}$$

$${\rm{Shell}}\,{\rm{mass}}=\frac{34}{66}\times 1.56\times {10}^{-19}\,{\rm{g}}=8.384\times {10}^{-20}{\rm{g}}$$

$${\rm{single}}\,{\rm{SPION}}\,{\rm{mass}}=1.56\times {10}^{-19}{\rm{g}}+0.8036\times {10}^{-19}{\rm{g}}=2.465\times {10}^{-19}{\rm{g}}$$”

should read:

“$${\rm{core}}\,{\rm{mass}}\,{\rm{of}}\,{\rm{single}}\,{\rm{SPION}}=5.024\frac{{\rm{g}}}{{10}^{21}\,{{\rm{nm}}}^{3}}\times 31.059\,{{\rm{nm}}}^{3}=1.560\times {10}^{-19}{\rm{g}}$$

$${\rm{Shell}}\,{\rm{mass}}=\frac{34}{66}{\rm{core}}\,{\rm{mass}}\,{\rm{of}}\,{\rm{single}}\,{\rm{SPION}}$$

$${\rm{S}}{\rm{h}}{\rm{e}}{\rm{l}}{\rm{l}}\,{\rm{m}}{\rm{a}}{\rm{s}}{\rm{s}}=\frac{34}{66}\times 1.56\times {10}^{-19}{\rm{g}}=8.036\times {10}^{-20}{\rm{g}}$$

$${\rm{single}}\,{\rm{SPION}}\,{\rm{mass}}=1.56\times {10}^{-19}{\rm{g}}+0.8036\times {10}^{-19}{\rm{g}}=2.364\times {10}^{-19}{\rm{g}}$$”

