# Supplementary material for: Optimization of Magneto-thermally Controlled Release Kinetics by Tuning of Magnetoliposome Composition and Structure
Source: Sci Rep. 2017 Aug 7;7:7474. doi: 10.1038/s41598-017-06980-9 (PMC5547053; doi:10.1038/s41598-017-06980-9)
Supplement: Supplementary file 1 — Supporting Information [file 41598_2017_6980_MOESM1_ESM.pdf]

Optimization of Magneto-thermally Controlled Release Kinetics by Tuning of Magnetoliposome Composition and Structure

Behzad Shirmardi Shaghasemi [a], Mudassar Mumtaz Virk [a], Erik Reimhult [a]*

[a] Institute for Biologically Inspired Materials, Department of Nanobiotechnology University of Natural Resources and Life Sciences, Muthgasse 11, 1190 Vienna, Austria
* Corresponding Author
E-mail address: erik.reimhult@boku.ac.at

Materials

Iron (0) pentacarbonyl 99.99% trace metal basis; dioctylether 99%; oleic acid technical grade 90%; Dopamine hydrochloride 98%; Sodium nitrite ACS reagent 97%; Sulfuric acid ACS reagent 95-98%; Palmitic acid >99%; Dicyclohexylcarbodiimide (DCC); N-Hydroxysuccinimide (NHS); Hydrochloric acid ACS reagent 37%; Calcein; Trizma base; Superdex 75; Triton X100 and all solvents were purchased from Sigma Aldrich and used as received without further purification. 1-palmitoyl-2-oleoyl-sn-glycero-3-phosphocholine (POPC), 1-myristoyl-2-palmitoyl-sn-glycero-3-phosphocholine (MPPC), 1,2-dipalmitoyl-sn-glycero-3-phosphocholine (DPPC) 1,2-distearoyl-sn-glycero-3-phosphocholine (DSPC), and 1,2- Dioleoyl-sn-Glycero-3-Phosphoethanolamine-N-[Methoxy(Polyethylene glycol)2000 Da] (Ammonium Salt) (PEG(2)-PE) were purchased from Avanti Polar Lipids (Alabama, USA) and used as received without further purification.

To study the stability of liposomes over time, magnetoliposome solutions (without calcein) were prepared (1mg/ml) with different ratios of iron oxide nanoparticle to lipid (2, 4, 6, 8 and 10wt%) and stored for 1 week at room temperature. Figure SI-1a shows photographs of the liposome dispersions. Aggregation was determined by naked eye and DLS. The samples were vortexed for 30 seconds before measurements at room temperature. The size of liposomes hosting 2wt% or 4wt% SPION remained almost the same after 1 week, but those which hosted more than 4wt% SPION precipitated and formed visible precipitations within 1 week. The samples with 6-8wt% SPION were still colored although they precipitated after a week; samples with 10wt% SPION precipitated completely within 1 week. Magnetoliposomes with 4wt% SPION were stored at room temperature for 11 months at the time of writing without observing visible precipitation or change to size distribution measured by DLS (Figure SI-1b). Size distribution of magnetoliposomes for different SPION concentrations measured one week after preparation using DLS is shown in Figure (SI 1c,d).

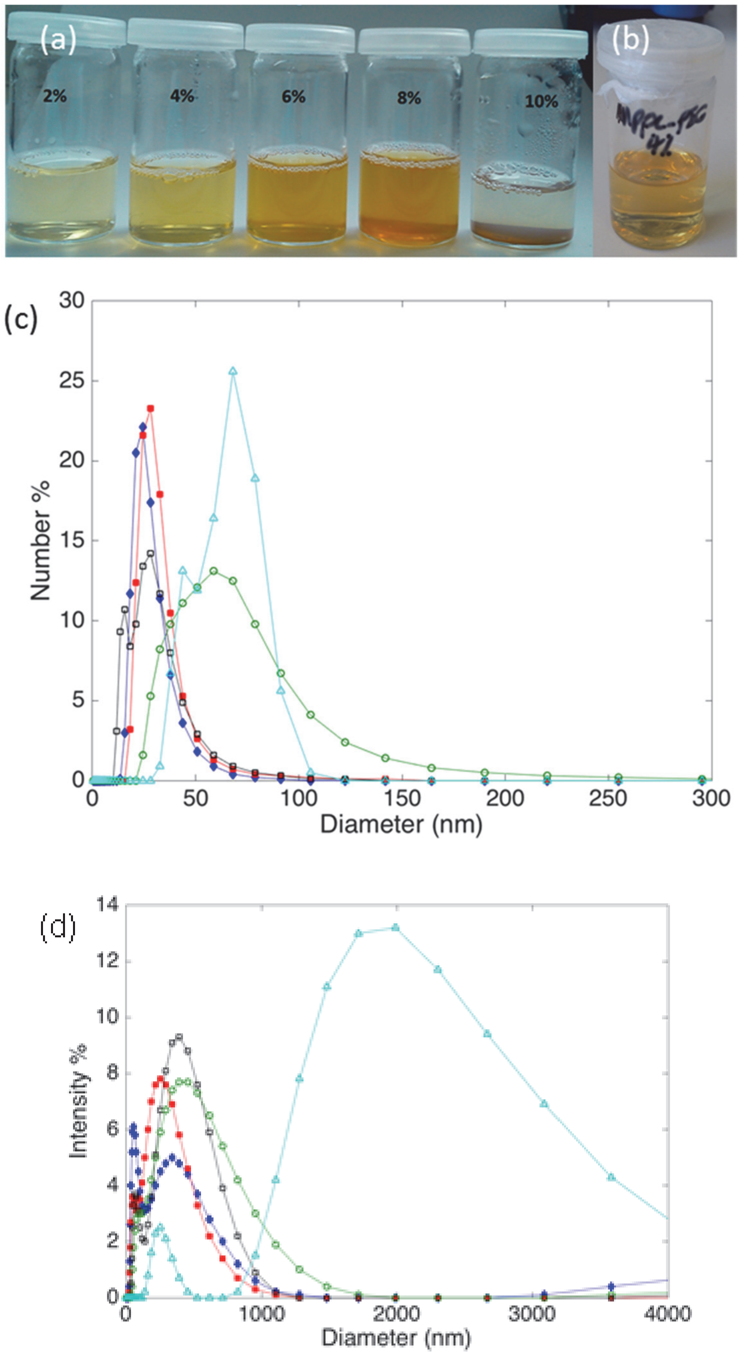

Figure SI-1 (a) PEGylated MPPC magnetoliposomes after 1 week observed optically for varied wt% of SPION incorporated. (b) Sample with 4wt% SPION after 11 months (c,d) Size distributions of MPPC magnetoliposomes with 2wt%(−◆−), 4wt%(−■−), 6wt%(−□−), 8wt%(−O−), 10wt%(−Δ−) SPION one week after their formation measured by DLS and displayed as the number weighted distributions (c) and intensity weighted distributions (d).

Trehalose-embedded liposomes and cryo-TEM were obtained for liposomes containing SPION. At low concentration, imaging liposomes such that SPION are visible is challenging due to the thin cross-section imaged and the low average number of SPION per liposome. The chance of detecting SPION increases at higher concentration of SPION. Liposomes imaged containing SPION showed them dispersed within the membrane without forming clusters, although they sometimes appeared to be in one part of the liposome membrane imaged in projection.

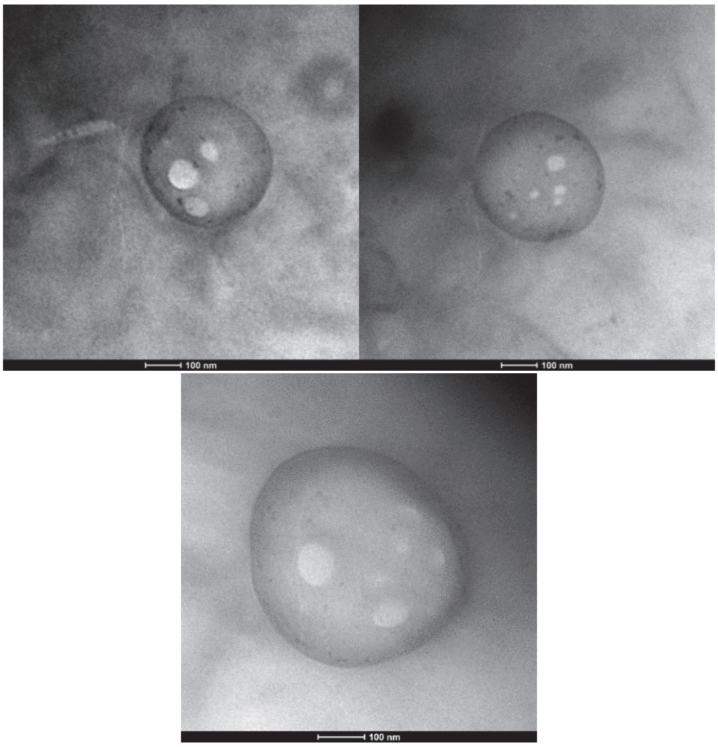

Figure SI-2 TEM micrographs of POPC liposomes loaded with 5 wt% 3.5 nm PNDA-SPION. Samples were prepared via solvent inversion and fixed in 1 %w/v trehalose by air drying. We thank Dr. Oliver Bixner for support with trehalose fixation and TEM.

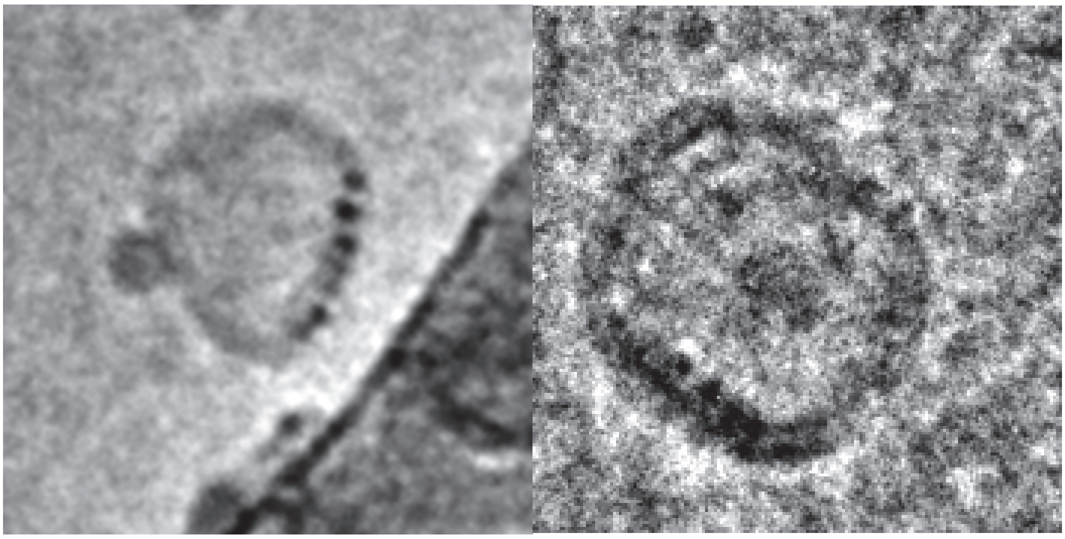

Figure SI-3 Cryo-TEM images of sonicated unilamellar liposomes containing 3.9nm/core diameter SPION. Cryo-TEM was performed on a 300 kV FEI Tecnai F30 Helium "Polara" TEM on plunge-frozen samples of 3.9wt% 4 nm PNDA-SPION in liposomes prepared by solvent inversion-sonication.

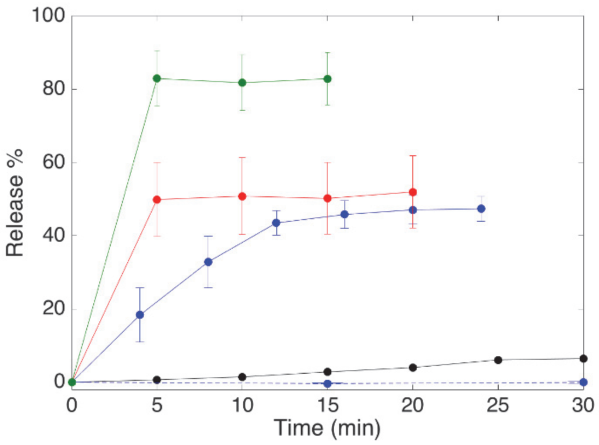

Figure SI-4 Release kinetics of DPPC magnetoliposomes with 2wt% SPION actuated with 4 min (blue) and 5 min (red) pulses. DPPC incorporated with 4wt% SPION actuated with 5 min pulses (green) and DPPC without SPION actuated for 5 min pulses (black).

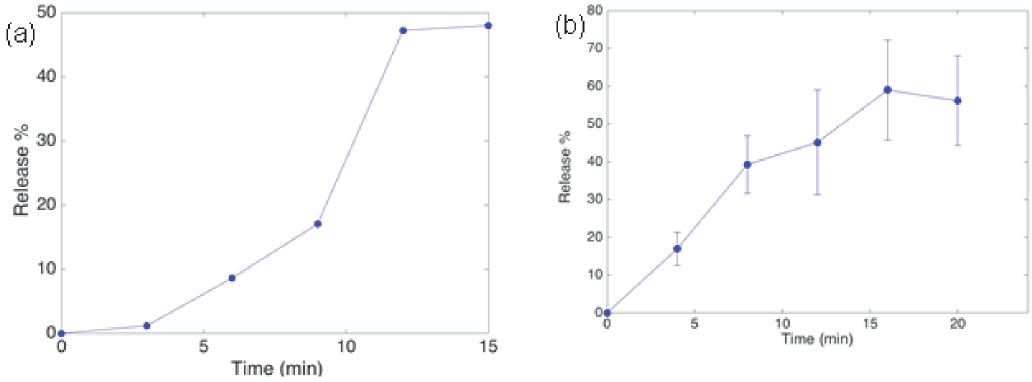

Fig SI-5 (a) Release kinetics of DPPC vesicles incorporating 6wt% SPION (4nm) exposed to 3min AMF pulses, (b) Release kinetics of DPPC vesicles incorporating 8wt% SPION (5nm) exposed to 4min AMF pulses.

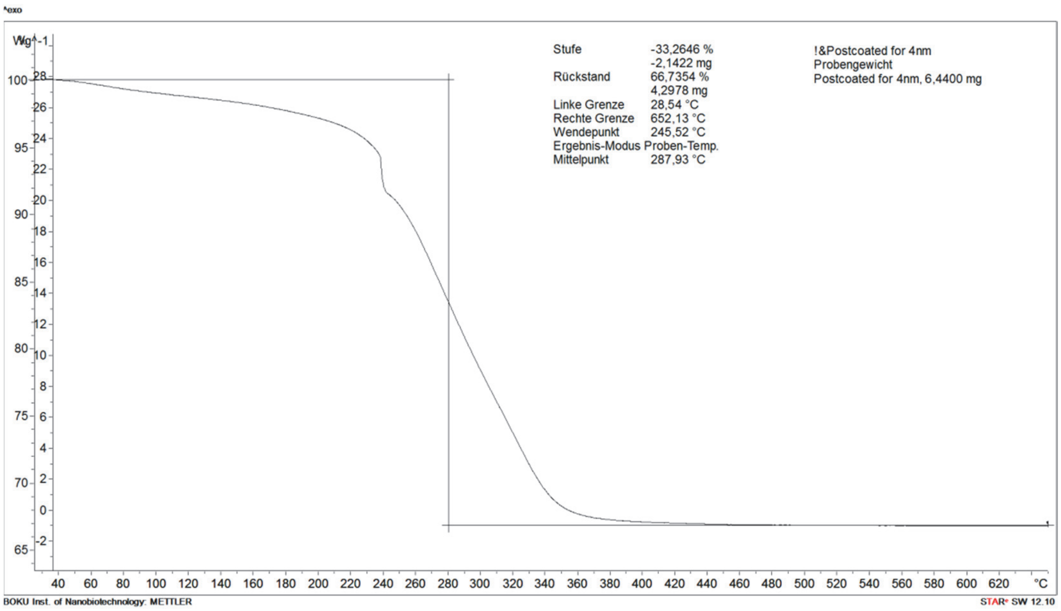

Figure SI-6 TGA graph of PNDA-capped SPION.

SI-7. The average mass of single SPION and number of SPION in Magnetoliposomes

The average number of SPION in magnetoliposomes was estimated by assuming that the particles are homogeneously distributed throughout the liposome distribution with no micellar aggregates present, as none were detected by DLS. The average radius of the liposomes was obtained by DLS. The mass of the shell of the SPION obtained from TGA.

Total number of lipids in 5 mg of lipids (N_{lip}) was calculated as:

$$\frac{0.005\ \text{g}}{733.5\ \text{g/mol}} = 6.816\times10^{-6}\ \text{mol}$$

$$N_{lip} = 6.816\times10^{-6}\times6.022\times10^{23} = 4.104\times10^{18}$$

where 733.5g/mol is the Mw of lipids.

Mass of single 3.9 nm SPION (m_{SPION}) was calculated as:

$$m_{SPION} = core\ mass + \text{shell mass}$$

$$core\ mass\ of\ single\ SPION = \rho\times\text{v}$$

$$\text{Volume of single SPION}\ \ \text{V} = \frac{4}{3}\pi R^3$$

where R is the radius of the SPION.

$$\text{Volume of single SPION}\ \ \text{V} = \frac{4}{3}\pi(1.95\ nm)^3 = 31.059\ nm^3$$

$$core\ mass\ of\ single\ SPION = 5.024\frac{\text{g}}{10^{21}nm^3}\times31.059\ \ nm^3 = 1.627\times10^{-19}g$$

$$Shell\ mass = \frac{34}{66}\ total\ mass\ of\ single\ SPION$$

$$Shell\ mass = \frac{34}{66}\times1.56\times10^{-19}g = 8.384\times10^{-20}g$$

$$single\ SPION\ mass = 1.56\times10^{-19}g + 0.8036\times10^{-19}g = 2.465\times10^{-19}g$$

Number of lipids per vesicle ($N_{lip/ves}$) was calculated as:

$$\text{Total surface area of liposome} = Surface\ are\ of\ outer\ layer + Surface\ area\ of\ inner\ layer$$

$$= 4\pi(R_V^2 + (R_V - t_b)^2)$$

where R_V is the radius of vesicle and t_b is the bilayer thickness which is assumed to be 4nm.

$$\text{Number of lipids per vesicle} = \frac{total\ surface\ area\ of\ vesicle}{head\ group\ area}$$

Average number of SPION per vesicle ($N_{SPION/ves}$) was calculated as:

$$N_{SPION/ves} = \frac{m_{SPIONtot}N_{lip/ves}}{m_{SPION}N_{lip}}$$

For example, in case of a MPPC 32.5 nm vesicle incorporated with 4wt% (0.2mg) SPION, average number of SPION per vesicle calculated using above equation is 2.1. Similarly, for 2wt% (0.1mg) SPION it yields 1.05 SPION per vesicle.

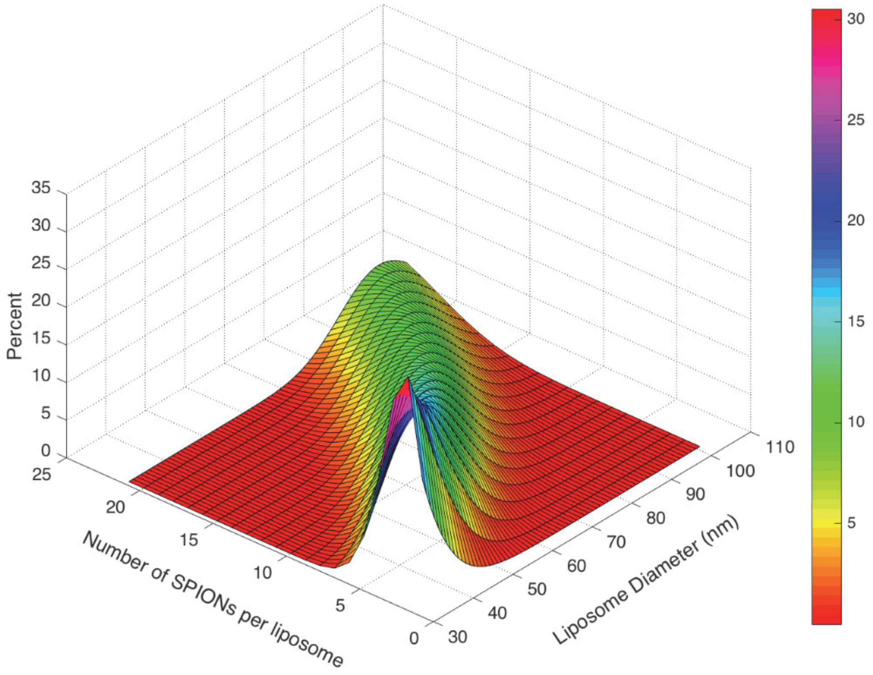

Figure SI-8 Surface plot of the distribution of liposomes with a certain number of SPION per liposome as function of liposome size when they are loaded with 4 wt% SPION (assuming a Poisson distribution of the nanoparticles among a monodisperse population of liposomes).

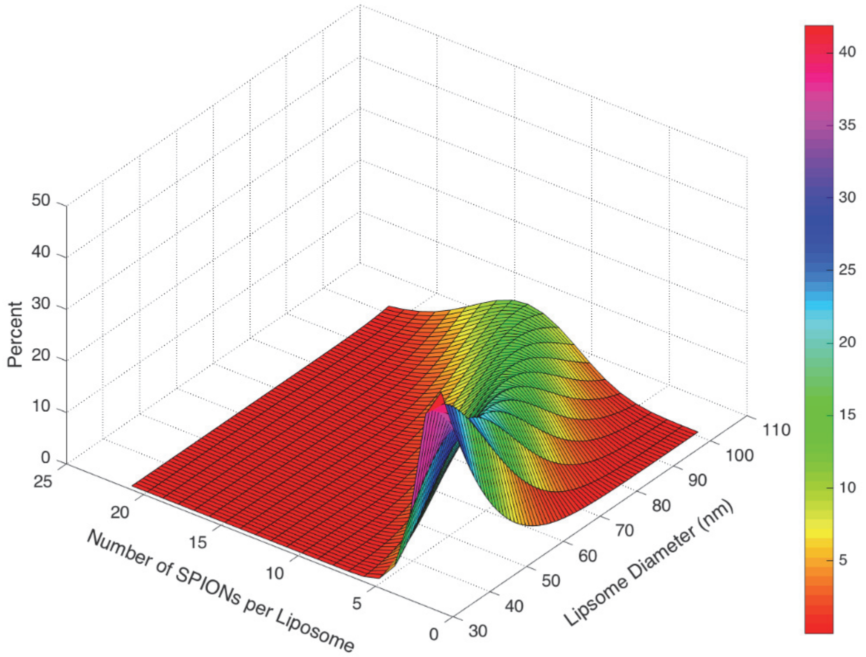

Figure SI-9 Surface plot of the distribution of liposomes with a certain number of SPION per liposome as function of liposome size when they are loaded with 2 wt% SPION (assuming a Poisson distribution of the nanoparticles among a monodisperse population of liposomes).

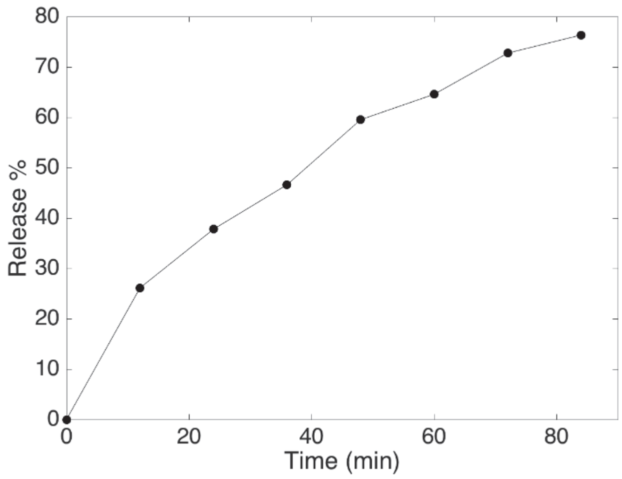

Figure SI-10 Release kinetics of DSPC magnetoliposomes with 4wt% SPION actuated for 12 min pulses followed by 5 min cooling down periods after each pulse.

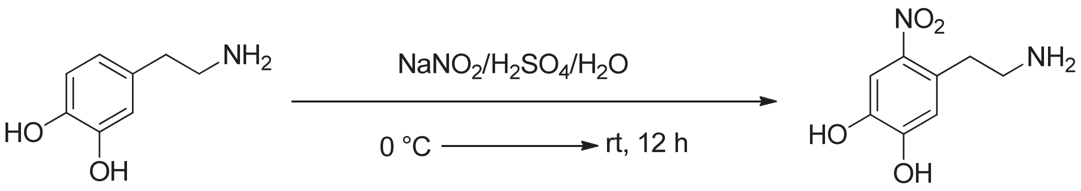

SI-11(a) Synthesis of Nitrodopamine (NDA).

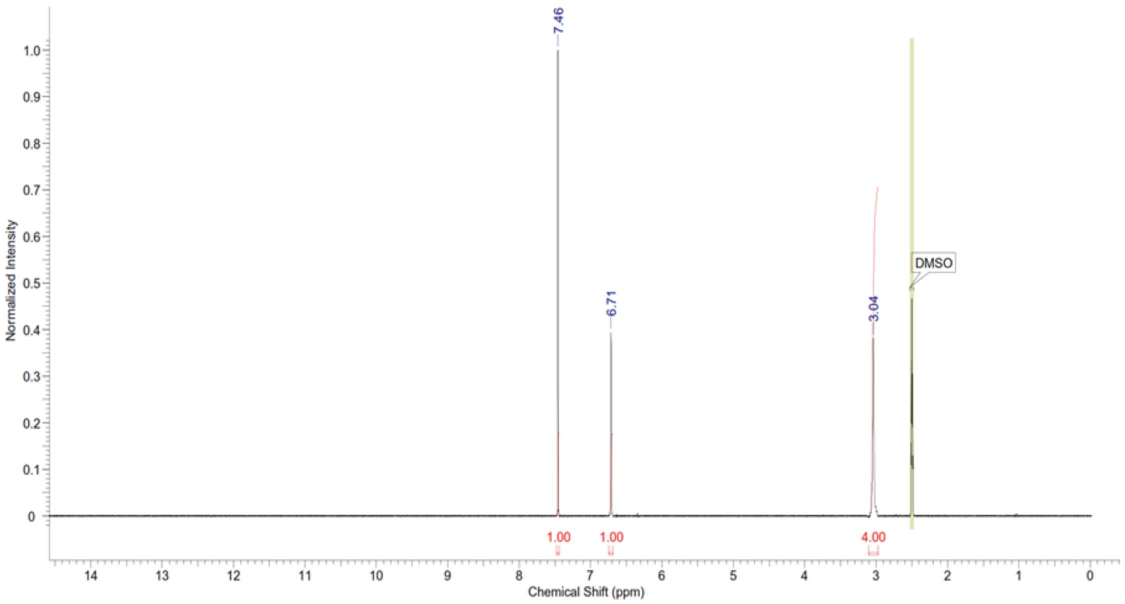

Figure SI-11(b) ^{1}HNMR spectrum of nitrodopamine (NDA).

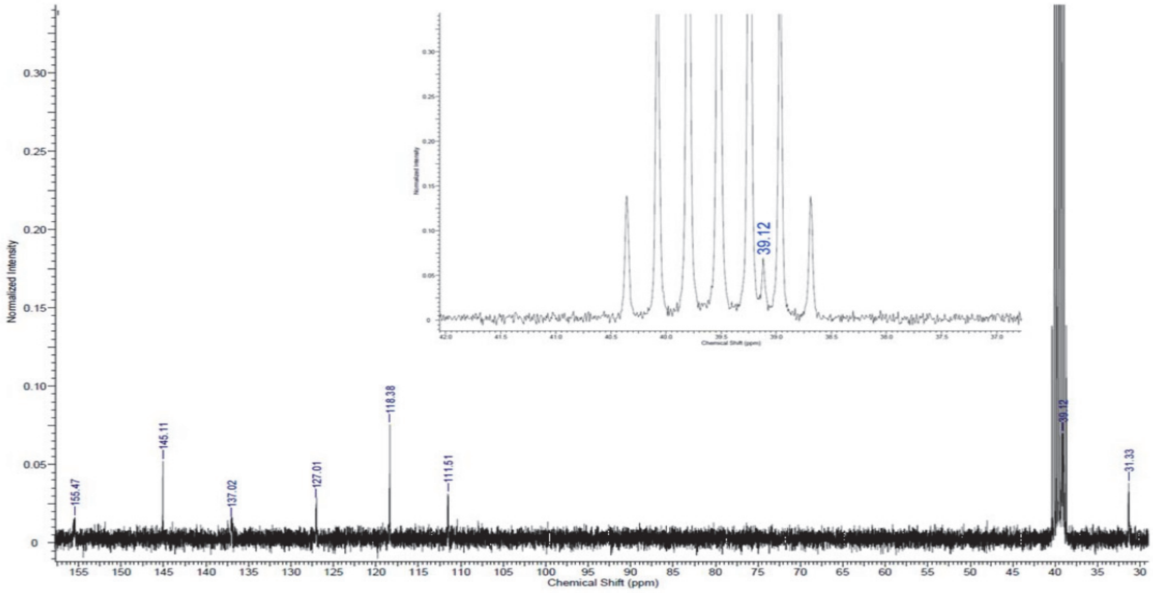

Figure SI-11(c) ^{13}CNMR spectrum of nitrodopamine (NDA).

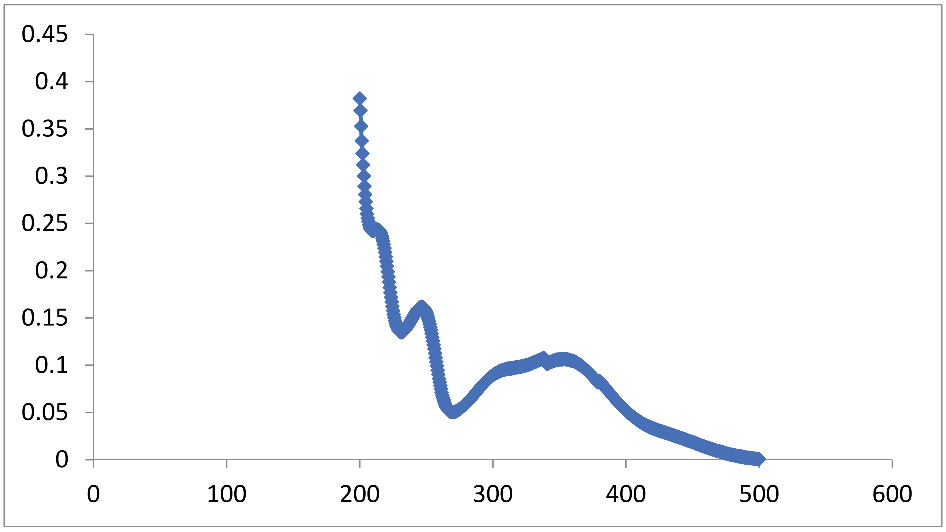

Figure SI-11(d) UV/VIS spectrum of nitrodopamine (NDA) in Water.

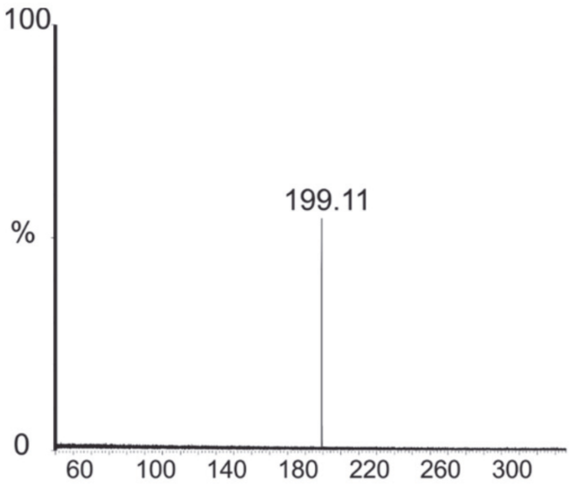

Figure SI-11(e) ESI-TOF-spectrum of nitrodopamine (NDA).

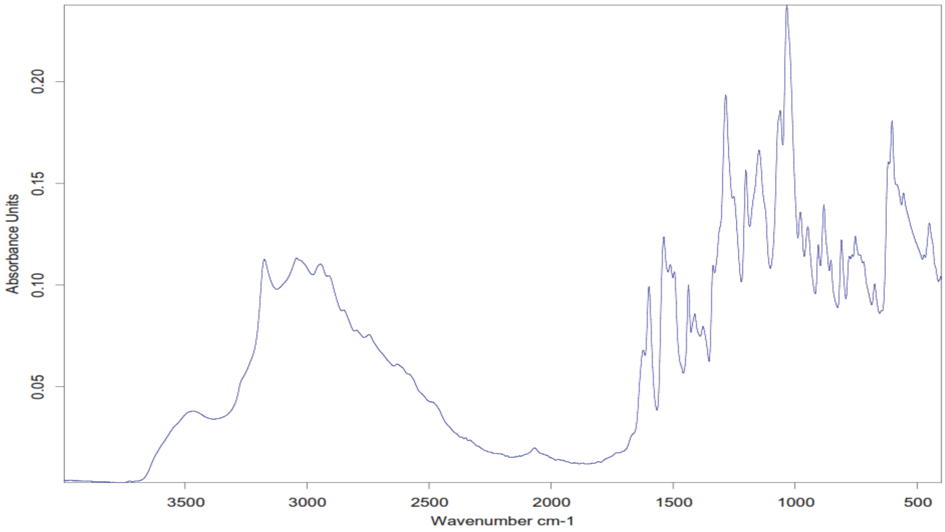

Figure SI-11(f) ATR-FTIR spectrum of nitrodopamine (NDA).

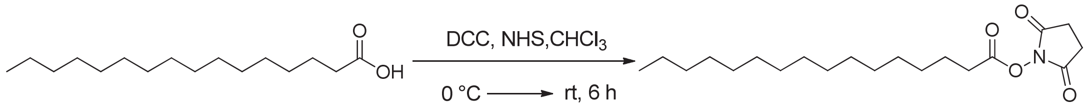

SI-12(a) Synthesis of palmityl-N-hydroxysuccinimide (PNHS).

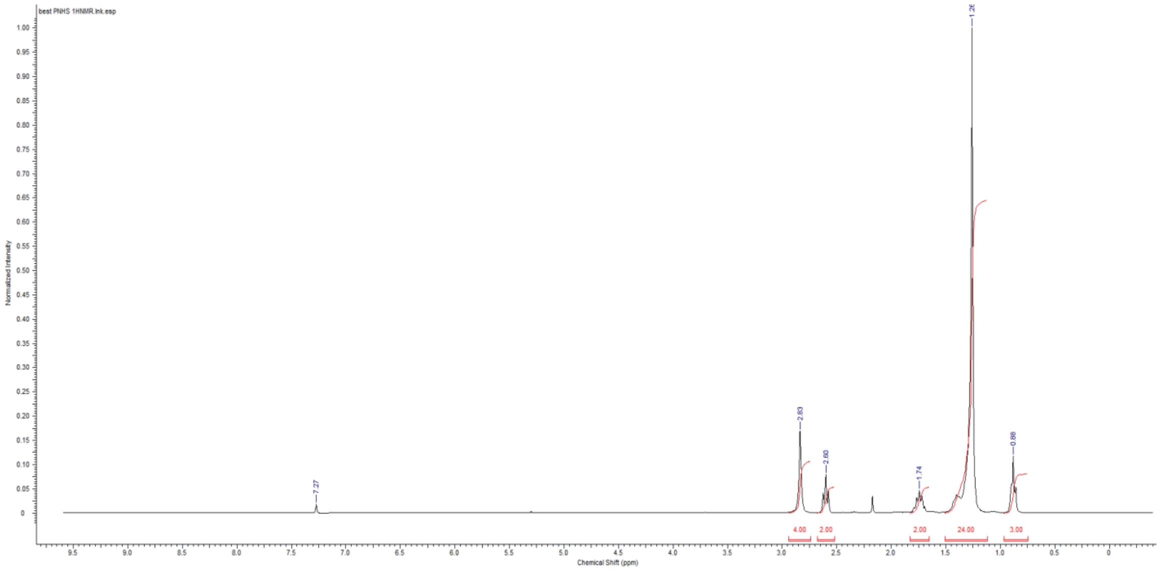

Figure SI-12(b) ^{1}HNMR spectrum of PNHS in $CDCl_3$.

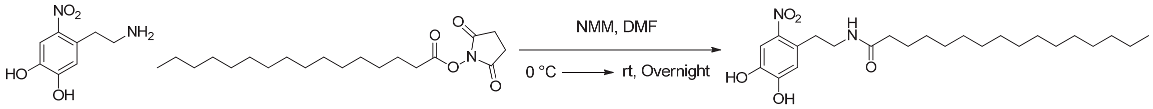

SI- 13(a) Synthesis of palmityl-nitrodopamide (PNDA).

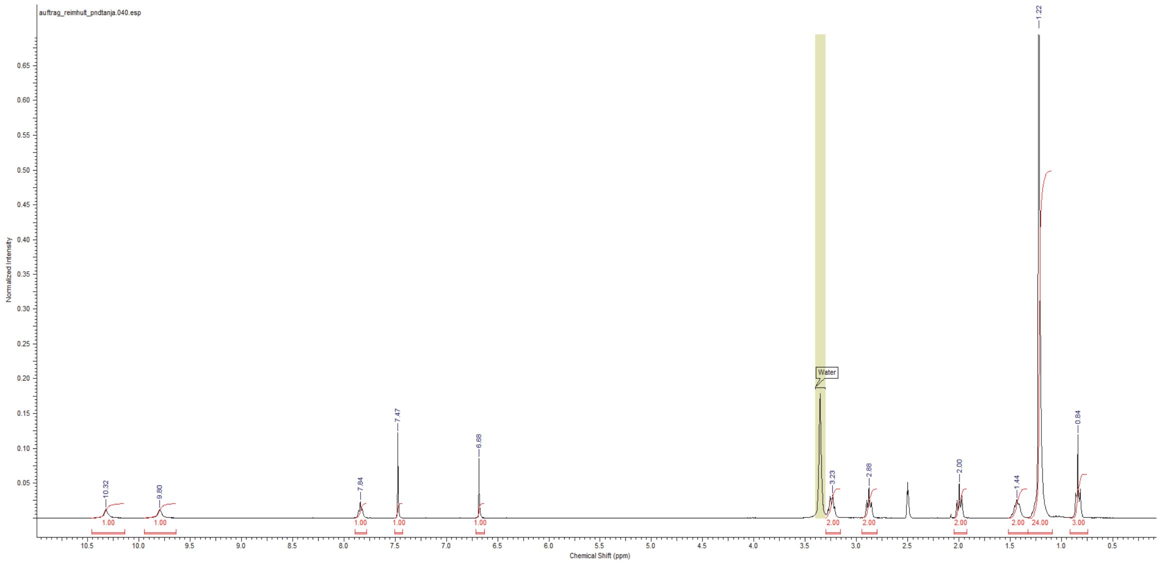

Figure SI-13(b) ^{1}HNMR spectrum of palmityl-nitrodopamide (PNDA) in DMSO-D_6.

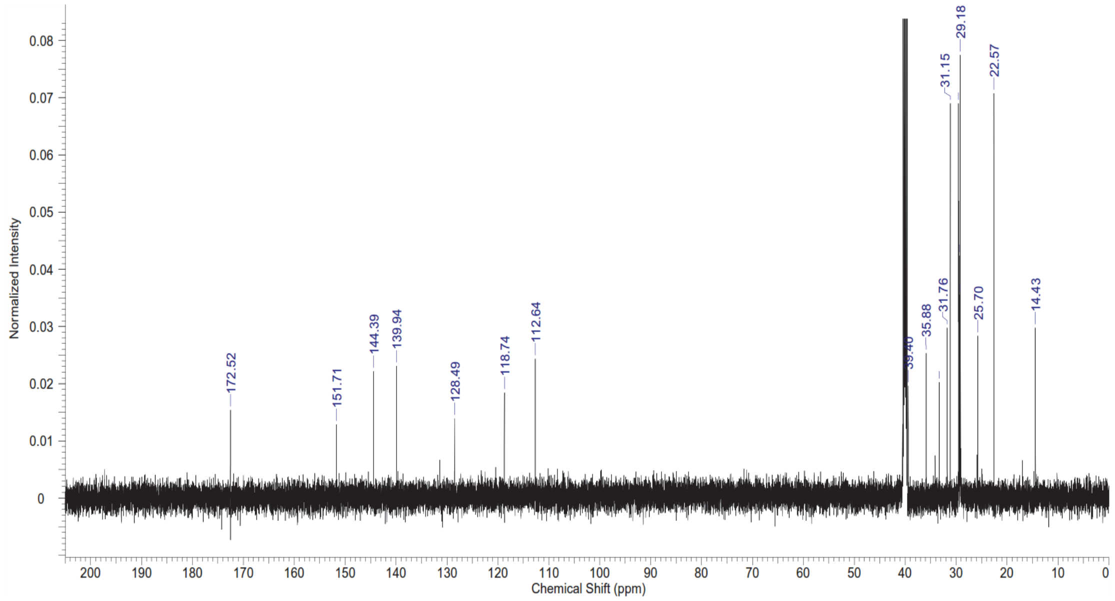

Figure SI-13(c) ^{13}CNMR spectrum of palmityl-nitrodopamide (PNDA) in DMSO- D_6.

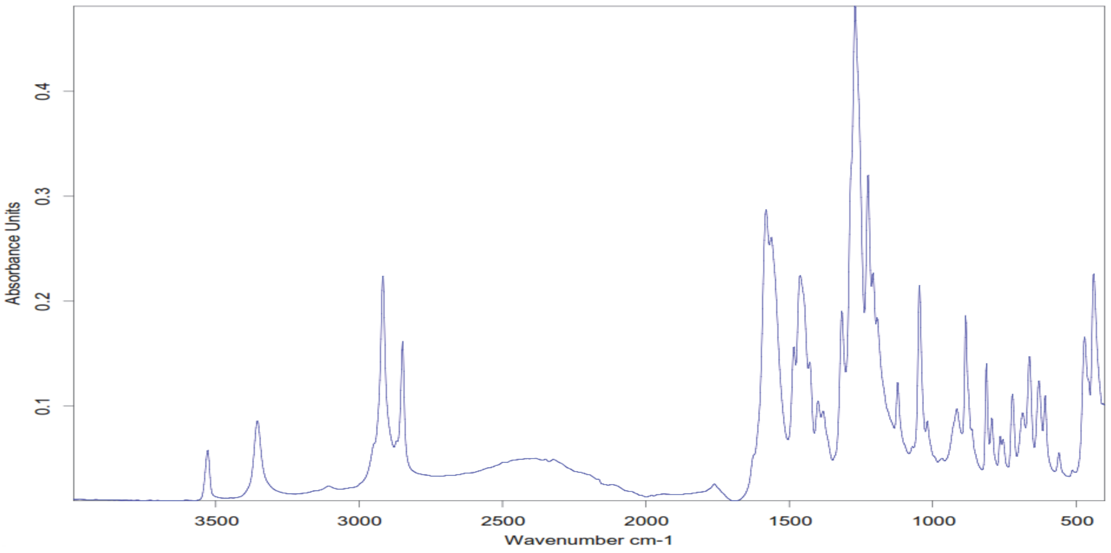

Figure SI-13(d) ATR-FTIR spectrum of palmityl-nitrodopamide (PNDA).

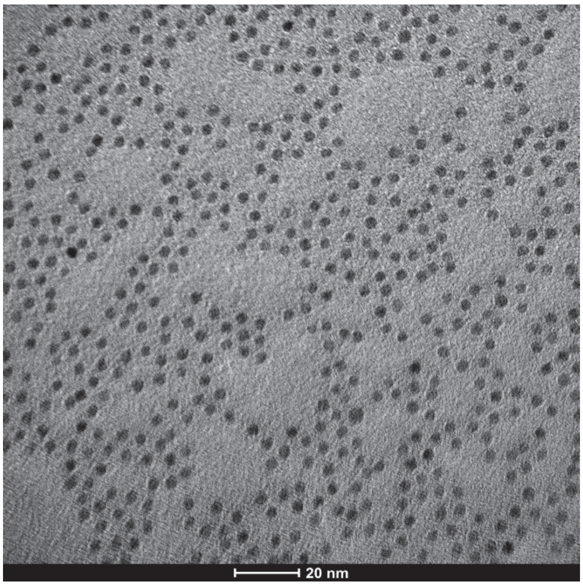

Figure SI-14(a) TEM image of PNDA-capped SPION in THF.

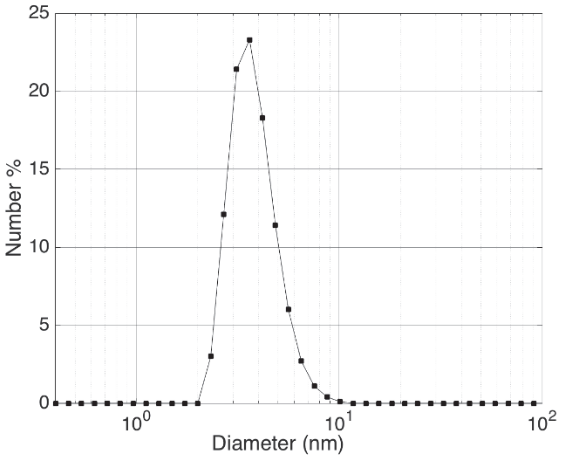

Figure SI-14(b) DLS graph of PNDA-capped SPION in THF.

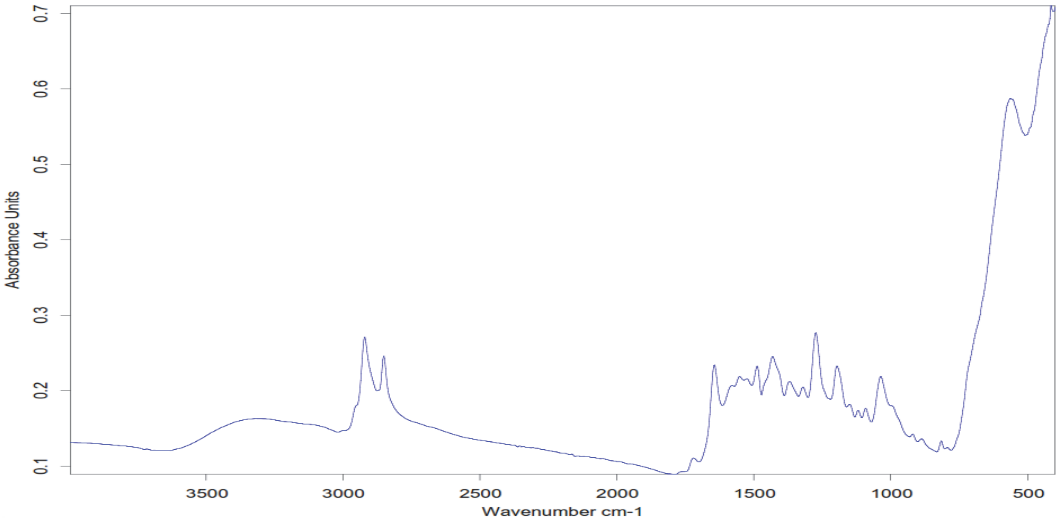

Figure SI-14(C) ATR-FTIR spectrum of PNDA-capped SPION.

A brief guide to interpreting the IR spectra: Physisorbed oleic acid has the following characteristic peaks in FTIR spectra: the peaks at 1703 cm^{-1} assigned to C=O stretch of hydrogen-bonded (dimeric) alkyl carboxylic acids. The OH in-plane and out-of-plane bending vibrations appears at 1430 and 965 cm^{-1}. These peaks are missing for the PNDA-capped SPION. The characteristic peak pf PNDA in FTIR spectra is the stretching vibration of C=O bond (amide) of PNDA at 1643 cm^{-1}. Fe−O lattice vibrations of magnetite are found at 580 cm^{-1} for OA-capped SPION and at 561 cm^{-1} for PNDA-capped SPION, respectively.

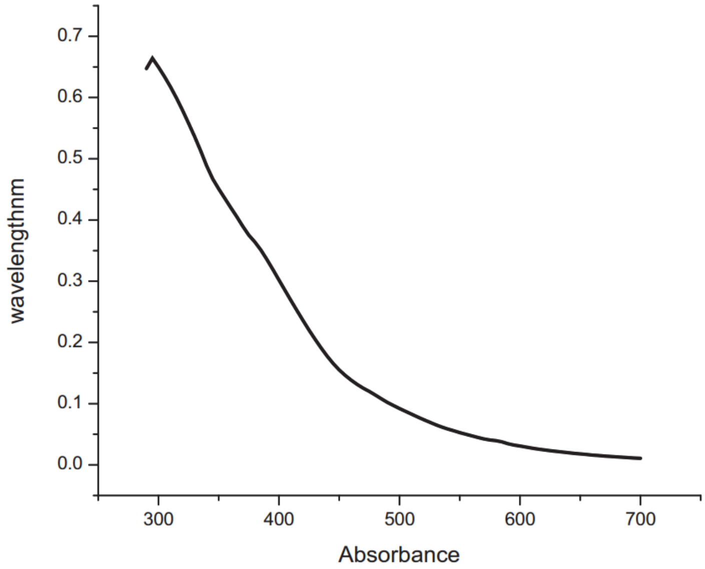

Figure SI-14(d) UV/vis spectrum of PNDA-capped SPION in THF.

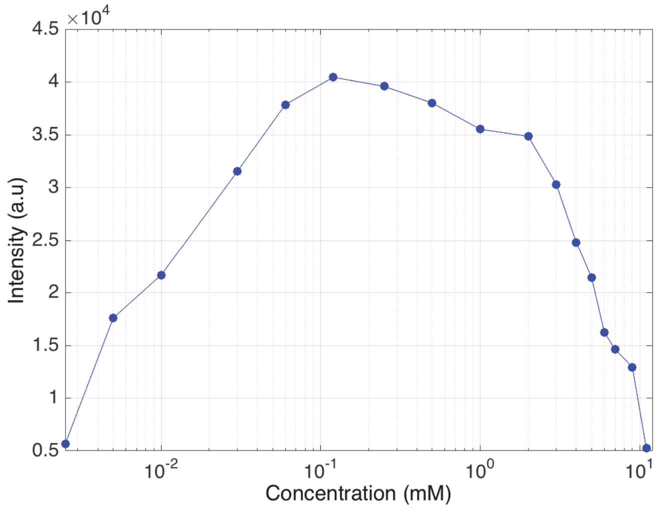

Figure SI-15 Intensity of calcein as function of concentration showing strong onset of self-quenching for concentrations >10^{-4} M.

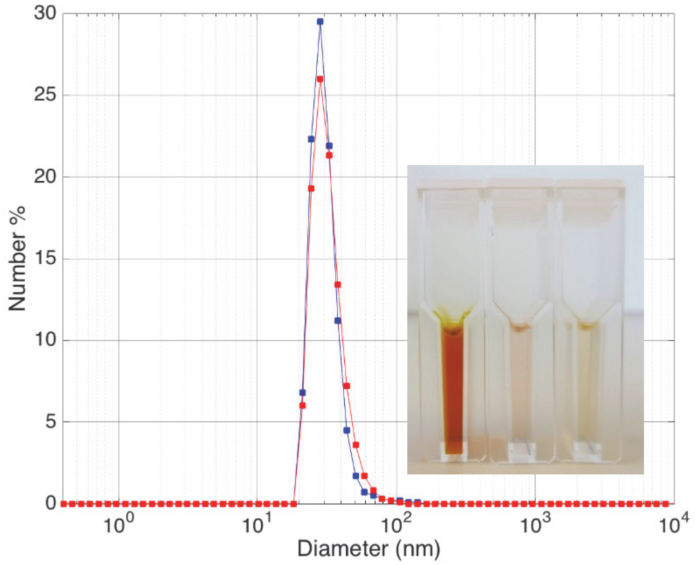

Figure SI-16 Size distribution of magnetoliposomes before (blue) and after (red) column separation. Inset shows calcein-loaded magnetoliposomes before (left) and after (middle) column separation and after magnetothermally triggered release (right).
